# Starazo triple switches – synthesis of unsymmetrical 1,3,5-tris(arylazo)benzenes

**DOI:** 10.3762/bjoc.16.4

**Published:** 2020-01-03

**Authors:** Andreas H Heindl, Hermann A Wegner

**Affiliations:** 1 Institute of Organic Chemistry, Justus Liebig University, Heinrich-Buff-Ring 17, 35392 Giessen, Germany; 2 Center for Material Research (LaMa), Justus Liebig University, Heinrich-Buff-Ring 16, 35392 Giessen, Germany

**Keywords:** multistate photoswitches, synthesis, tris(arylazo)benzene

## Abstract

Multistate switches allow to drastically increase the information storage capacity and complexity of smart materials. In this context, unsymmetrical 1,3,5-tris(arylazo)benzenes – ‘starazos’ – which merge three photoswitches on one benzene ring, were successfully prepared. Two different synthetic strategies, one based on Baeyer–Mills reactions and the other based on Pd-catalyzed coupling reactions of arylhydrazides and aryl halides, followed by oxidation, were investigated. The Pd-catalyzed route efficiently led to the target compounds, unsymmetrical tris(arylazo)benzenes. These triple switches were preliminarily characterized in terms of their isomerization behavior using UV–vis and ^1^H NMR spectroscopy. The efficient synthesis of this new class of unsymmetrical tris(arylazo)benzenes opened new avenues to novel multistate switching materials.

## Introduction

The reversible photochemically induced structural change of azobenzenes (ABs) opens various ways towards systems manipulations on the molecular level [1–2]. Upon irradiation with UV light (ca. 350 nm), the thermodynamically more stable (*E*)-AB isomerizes to the higher-energy *Z*-isomer [3]. The isomerization can be reversed either by irradiation with visible light (ca. 450 nm) or thermally, upon heating. The thermal back-isomerization rate can be controlled by various factors: Functional group substitutions on the phenyl rings determine the thermal half-lives of (*Z*)-ABs [1]. For example, *o*-fluoro substitution prolongs the thermal stability up to years [4], while electron-donating groups attached to the different phenyl rings decrease the thermal half-lives below the time scale of seconds. Even subtle interactions, such as London dispersion in alkyl-substituted ABs, can have a significant influence on their isomerization properties [3–4]. Also, the incorporation of AB units into cyclic [5] or macrocyclic structures can control the switching, depending, i.a., on symmetry and ring strain [6–9]. By combining these approaches, half-lives can be tuned from milliseconds to years.

**Figure 1:**
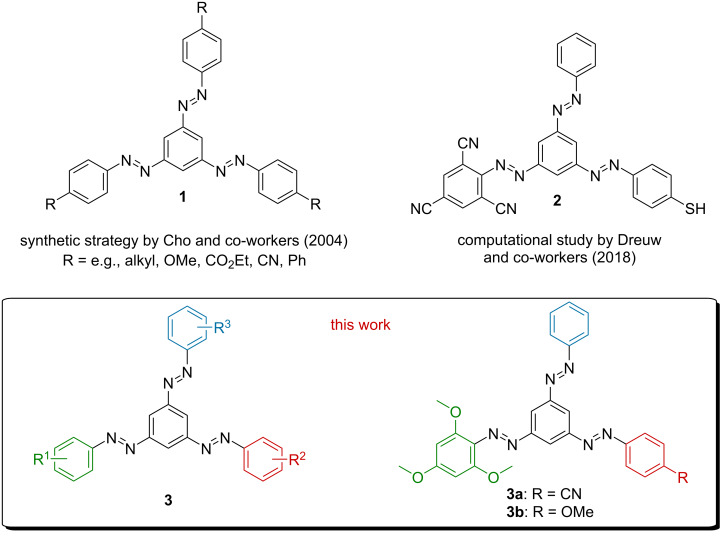
1,3,5-Tris(arylazo)benzenes as individually switchable multistate photoswitches: previous [11–12] and present work.

The incorporation of multiple AB units into one molecule allows to access multiple states upon isomerization, which dramatically increases the potential for information storage using this photoswitch. Ideally, one molecule can be treated with multiple different inputs, leading to defined, detectable outputs. An example of such compounds are 1,3,5-tris(arylazo)benzenes – ‘starazos’ – introduced by Cho and co-workers in 2004 (Figure 1) [10]. Despite their successful synthesis, using Pd-catalyzed coupling reactions of aryl halides and arylhydrazides [11] followed by Cu(I)-mediated oxidation, the photochemical properties of such compounds have not been studied yet. Going one step further, this type of compounds could be substituted in an unsymmetrical way with different azo units, which allowed individual switching using light of different wavelengths [12]. These compounds were then investigated theoretically by Dreuw and co-workers, who suggested tris(arylazo)benzene **2** (Figure 1) to feature spectrally separated absorption bands for each AB branch. The authors could show that the excited states of the AB branches in tris(arylazo)benzenes were electronically decoupled, despite the spatial overlap. Hence, only four phenyl rings were sufficient for the construction of three individually photoisomerizable azo units.

Motivated by the promising theoretical results by Dreuw and co-workers [12], an efficient synthetic strategy for asymmetric tris(arylazo)benzenes **3** was developed. With an efficient preparative access, this highly interesting class of multistate photoswitches would be accessible for detailed spectroscopic investigations, leading to fundamental insights applicable to the design of intelligent photoresponsive materials.

## Results and Discussion

**Scheme 1:**
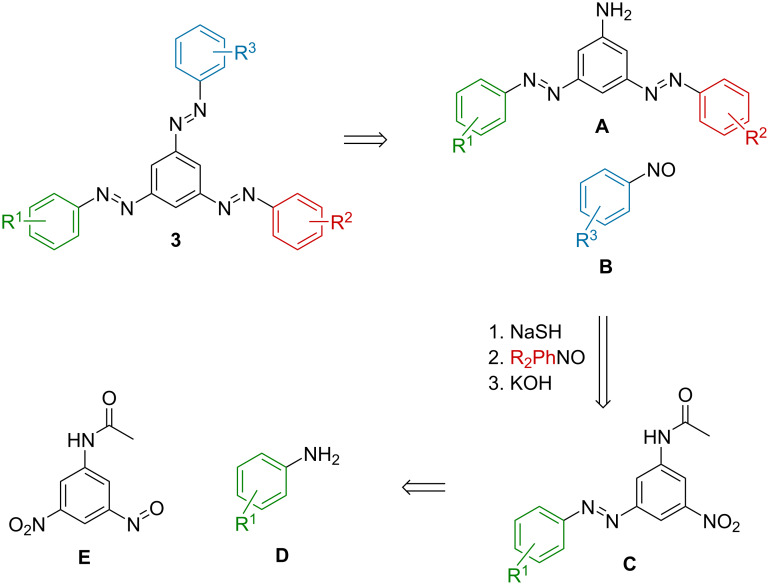
Synthetic strategy towards 1,3,5-tris(arylazo)benzenes **3** based on consecutive Baeyer–Mills reactions.

Initially, two different synthetic strategies were evaluated for the preparation of tris(arylazo)benzenes **3**. The first one relied on the consecutive condensation of anilines with nitroso compounds, Baeyer–Mills reactions [13]. With a suitable protecting group strategy, the selective construction of the individual AB branches should be achievable (Scheme 1). Starting from 3,5-disubstituted nitrosobenzene **E**, consecutive Bayer–Mills and deprotection reactions would lead to the target compound **3** in five steps. Furthermore, by using nitrosoarene **E**, the selective installment of the amine groups, e.g., via acetamide- and nitro group-carrying intermediates, could be achieved [14–16]. Such a strategy had already been used successfully in previous syntheses of *o-*, *p-*, and *m-*bis(arylazo)benzenes in our laboratory [14]. However, multiple protection/deprotection steps lowered the atom economy and increased the step count of this strategy.

**Scheme 2:**
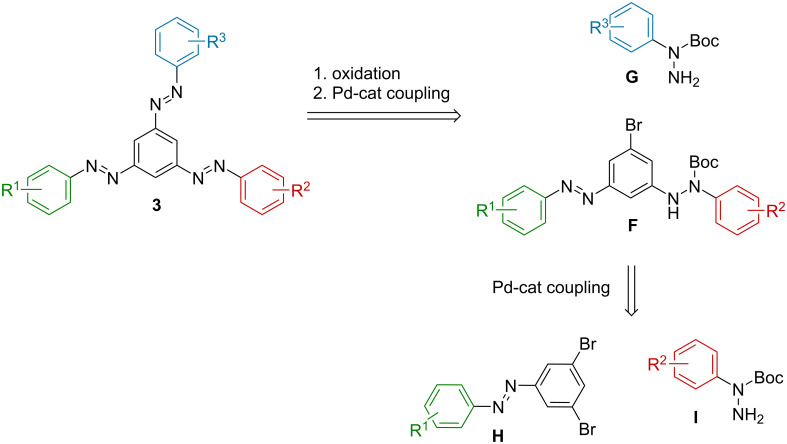
Preparation of tris(arylazo)benzenes **3** by Pd-catalyzed cross-coupling reactions of *N*-Boc-arylhydrazides and bromo-substituted ABs, followed by oxidation.

The second approach towards tris(arylazo)benzenes **3** relied on Pd-catalyzed coupling reactions and Cu(I) oxidation, as presented by Cho and co-workers (Scheme 2) [10]. This route offers the advantage that the preparation of a wide variety of *N*-(*tert*-butoxycarbonyl)phenylhydrazides has already been reported [17–19]. Additionally, starting from easily accessible 3,5-dibromoazobenzenes **H**, only two consecutive coupling reactions, followed by oxidation, would be required to obtain the target starazo **3**. However, selectivity might be problematic in the first coupling reaction, which would lead to lower yields of the desired monocoupled intermediates.

**Scheme 3:**
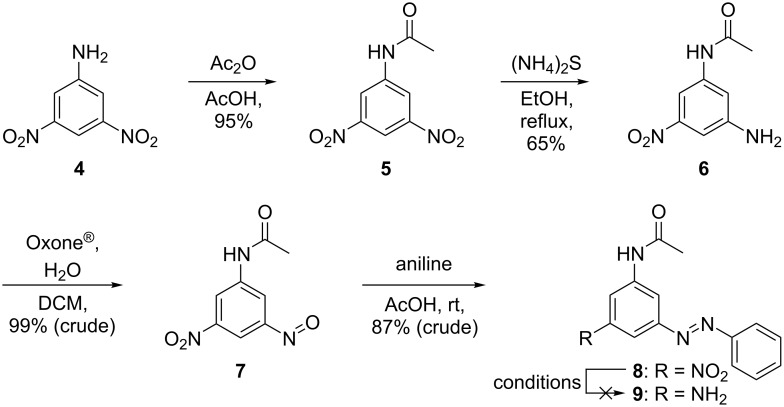
Synthesis of azobenzene building block **8**.

As target compounds, tris(arylazo)benzenes (Figure 1) with an electron-neutral, electron-donating, and electron-poor (**3a**), or with two different electron-donating (**3b**) azobenzene fragments were envisioned to test both synthetic strategies. First, the Bayer–Mills route was followed. The azobenzene building block **8**, with an unsubstituted phenyl ring and two orthogonal nitrogen substituents in the 3- and 5-position (Scheme 3), was prepared. The synthesis commenced with the acetylation of 3,5-dinitroaniline (**4**) in 95% yield, followed by the selective reduction of one nitro group using an aqueous ammonium sulfide solution to furnish aniline **6** in 65% yield [20]. After oxidation of **6** to its nitroso analogue **7** [21], a Baeyer–Mills reaction with aniline yielded the targeted azobenzene building block **8** in 87% yield (i.e., 53% yield over four steps).

After the successful synthesis of (*E*)-*N*-(3-nitro-5-(phenyldiazenyl)phenyl)acetamide (**8**), the reduction of the nitro group was attempted. However, the literature-known procedure of using sodium hydrosulfide did not yield the desired (*E*)-*N*-(3-amino-5-(phenyldiazenyl)phenyl)acetamide (**9**). Alternative reduction attempts, such as using Pd/C-catalyzed reduction by H_2_ in different solvents, SnCl_2_, or iron under acidic conditions were not successful either. In all cases, the reactions produced complicated mixtures, and the target compound could neither be identified nor isolated. At this stage, the second strategy via Pd-catalyzed coupling reaction was assayed [10–11].

**Scheme 4:**
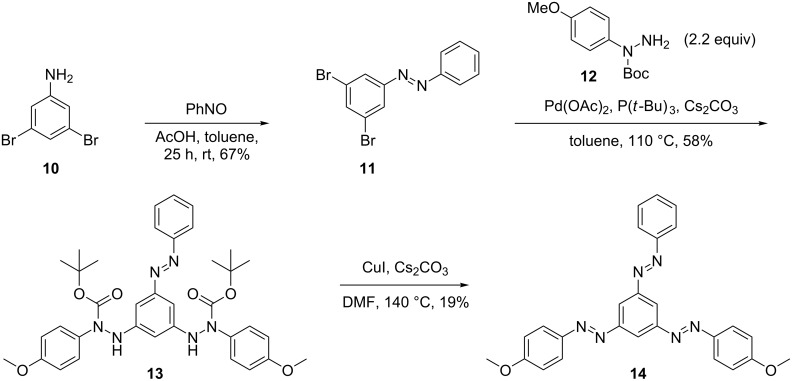
Synthesis of 1,3-bis(4-methoxyphenylazo)-5-phenylazobenzene (**14**).

In order to investigate the applicability of the Pd-catalyzed route to *C*_2_-symmetric compounds, 1,3-bis(4-methoxyphenylazo)-5-phenylazobenzene (**14**) was targeted. First, via coupling of 3,5-dibromoazobenzene (**11**) [22–23] using 2.2 equiv of 4-methoxyphenyl-*N*-Boc-hydrazide (**12**, Scheme 4). The reaction yielded the intermediate **13** in 58% yield, using slightly modified conditions compared to the literature protocol [10]. The CuI-mediated oxidation of the bis(arylhydrazide) **13** afforded the desired tris(arylazo)benzene **14** in only 19% yield, which was significantly lower compared to the literature-known data for symmetric tris(arylazo)benzenes **1**.

**Scheme 5:**
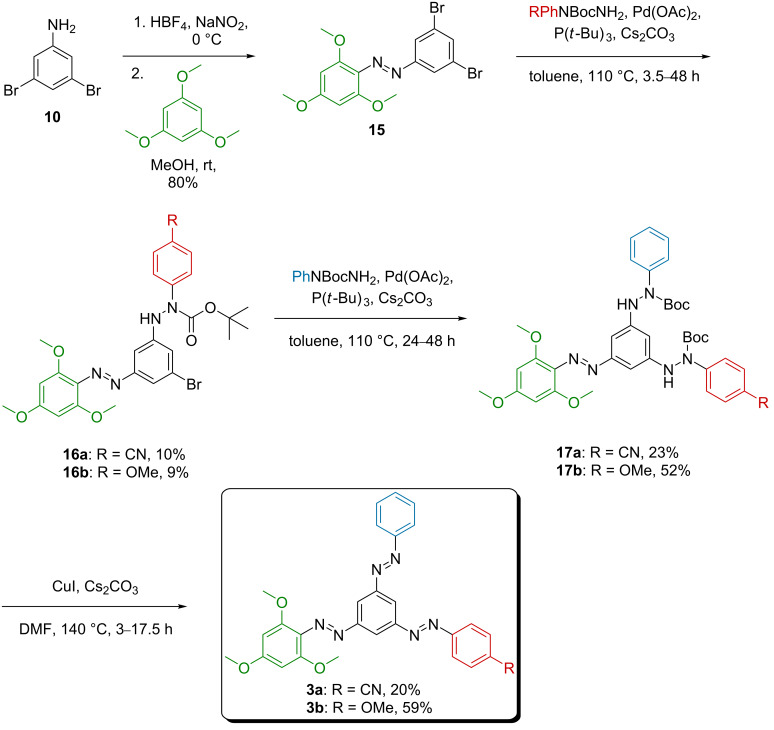
Synthesis of unsymmetrical tris(arylazo)benzenes **3a**/**3b** using Pd-catalyzed coupling reactions and CuI-mediated oxidation as key steps.

**Scheme 6:**
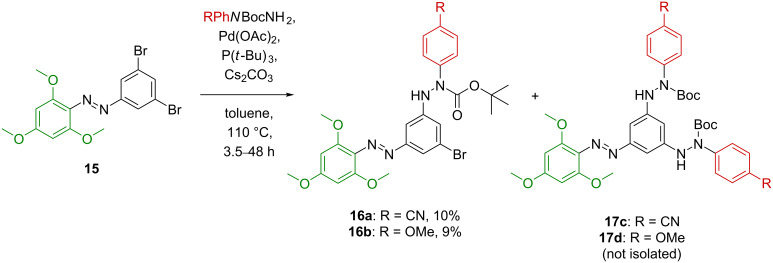
Coupling reactions of **15** with the corresponding arylhydrazides to access monocoupled (**16a**/**16b**) and biscoupled compounds (**17c**/**17d**) as major products.

After this preliminary test, the synthesis of unsymmetrical tris(arylazo)benzenes **3a**/**3b** was attempted (Scheme 5). In order to install the first, electron-rich 2,4,6-trimethoxyazobenzene fragment, 3,5-dibromoaniline (**10**) was diazotized and treated with 1,3,5-trimethoxybenzene to afford (*E*)-1-(3,5-dibromophenyl)-2-(2,4,6-trimethoxyphenyl)diazene (**15**) in high yield. Unfortunately, the following monocoupling step using (4-cyanophenyl)- or (4-methoxyphenyl)arylhydrazides turned out to be rather ineffective. The desired monocoupling products **16a**/**16b** could only be isolated in ca. 10% yield. It was found that the second coupling, leading to the bis(arylhydrazide) products **17c**/**17d**, showed higher reaction rates (Scheme 6). For the reaction of **15** with (4-methoxyphenyl)-*N*-Boc-hydrazide, 21% of the starting material **15** were recovered, indicating 79% conversion.

Nevertheless, both AB **15** as well as the arylhydrazides could be prepared on a multigram scale, which allowed to access the desired monocoupling products **16a**/**16b** on a scale of several hundred milligrams. Their ^1^H NMR spectra indicated the presence of mixtures of several isomers, not only *E*- and Z-configuration of the AB, but also different configurations of the Boc groups. However, the coupling products could be unequivocally identified by high-resolution mass spectrometry. Next, **16a**/**16b** were coupled with *N*-Boc-*N*-phenylhydrazine to afford the corresponding tris(arylazo)benzene precursors **17a** in 23% and **17b** in 52% yield, respectively (Scheme 5). Again, like for the first coupling reaction, it was not possible to isolate the products as uniform isomers using chromatographic methods. Hence, the tris(arylazo)benzene precursors **17a**/**17b** were used without further purification.

Initially, a coupling reaction of *N*-Boc-*N*-phenylhydrazine with AB **15** was attempted. However, with this inversed reaction sequence, inseparable mixtures of the desired coupling product and the AB starting material **15** were obtained. Hence, coupling of the electron-poor and electron-rich arylhydrazides was performed first to allow the isolation of **16a**/**16b** by chromatographic methods.

Finally, the target tris(arylazo)benzenes **3a**/**3b** were obtained by oxidation of the precursors **17a**/**17b** using CuI under basic conditions in DMF at elevated temperature (Scheme 5). For the cyano-substituted derivative **3a**, a yield of 20% was achieved, whereas the methoxy derivative **3b** could be isolated in a good yield of 59%.

**Figure 2:**
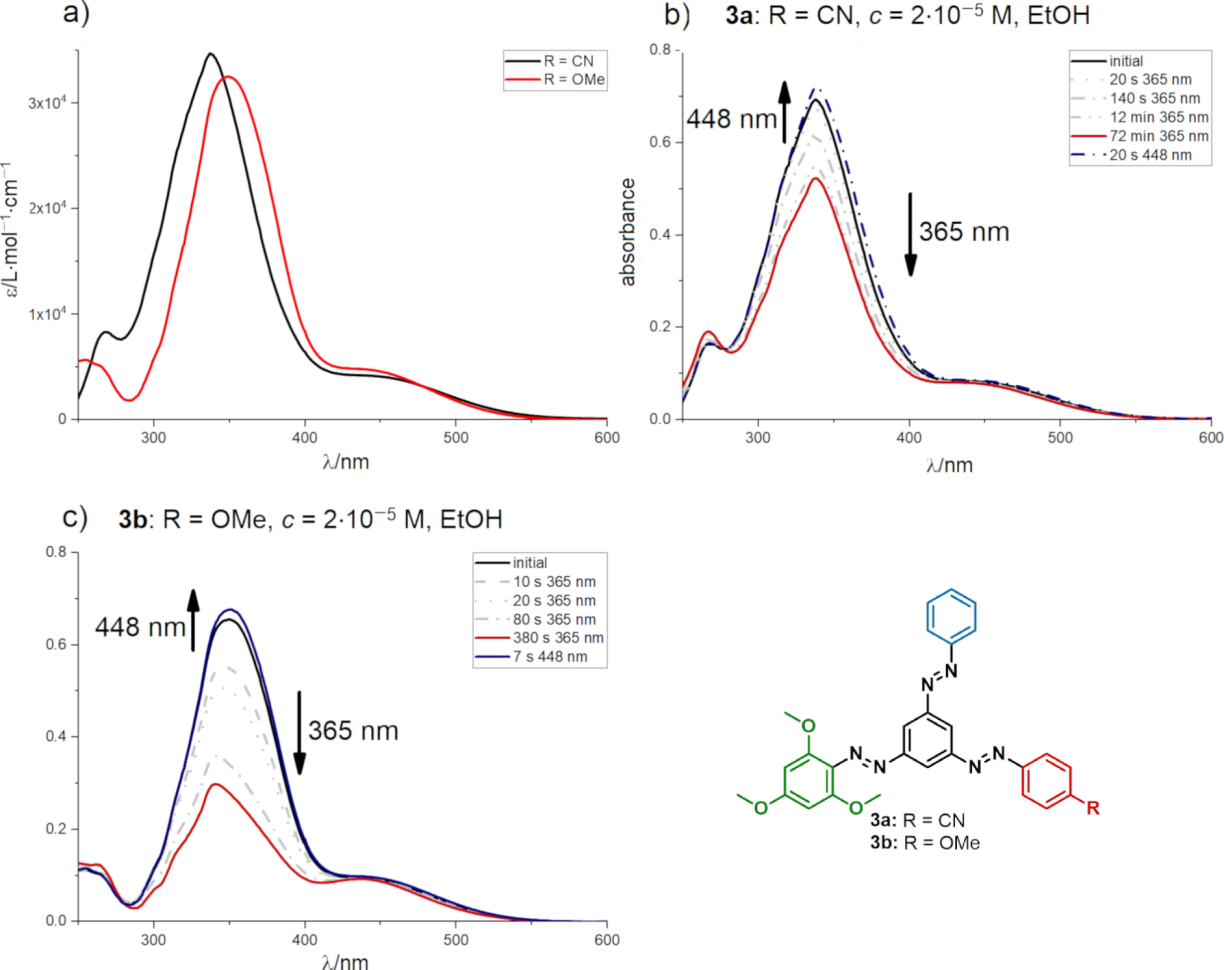
a) UV–vis spectra of tris(arylazo)benzenes **3a**/**3b** in ethanol. b) Reversible photoisomerization of **3a** and c) **3b** using light of 365 nm wavelength to induce *E*→*Z* isomerization and of 448 nm wavelength for photochemical back-conversion.

Having both tris(arylazo)benzenes **3a**/**3b** in hand, UV–vis spectroscopy was used for preliminary investigations on the photophysical properties of the molecular triple switches **3**. Both tris(arylazo)benzenes **3a** and **3b** showed characteristic UV–vis spectra of ABs with strong π–π* absorption, having their absorption maxima at 337 nm (**3a**) and 349 nm (**3b**), respectively (Figure 2a). In contrast to AB, the π–π* and n–π* bands of both compounds overlapped, forming shoulders at ca. 450 nm. The samples were irradiated at 365 nm with a high-power LED (see Supporting Information File 1 for specifications) to induce *E*-to-*Z* photoisomerization.

For the cyano-substituted tris(arylazo)benzene species **3a**, the π→π^*^ absorption band decreased only to a relatively small extent, while for starazo **3b**, the expected switching behavior was more pronounced. Furthermore, the irradiation time for reaching the photostationary state (PSS) of **3a** was significantly longer compared to **3b** or other AB derivatives. Afterwards, the solutions were irradiated with light of 448 nm to photochemically induce *Z*-to-*E* isomerization. In both cases, spectra of PSSs with slightly higher *E*/*Z* ratio could be reached (Figure 2b and Figure 2c). This is consistent with the fact that the tris(arylazo)benzenes **3a**/**3b** were isolated as mixtures of the all-*E*-isomers including small amounts of other photoisomers after the synthesis. Furthermore, the all-*E* states could be reached after only 20 and 7 s of irradiation, respectively, which was significantly faster than the *E*→*Z* photoisomerization. All in all, the irradiation experiments revealed that the methoxy-substituted derivative **3b** shows reversible photoisomerization, while cyano-substituted tris(arylazo)benzene **3a** could only be marginally isomerized.

To get deeper insight into the photoisomerization, ^1^H NMR spectroscopy was applied to monitor the isomerization process. For both **3a** and **3b**, complex spectra were obtained after irradiation at 365 nm (see Figure S2 and Figure S3, Supporting Information File 1). Hence, a high number of photoisomers was generated, which indicated that selective photoisomerization was not possible in both cases under the applied conditions. After irradiating the samples with light of 448 nm wavelength and keeping them in the dark at room temperature, the initial spectra could be restored.

## Conclusion

In summary, 1,3,5-tris(arylazo)benzenes **3a**/**3b** were successfully synthesized using Pd-catalyzed coupling reactions of arylhydrazides and aryl bromides followed by CuI-mediated oxidation as key steps. Although the low yield of the first coupling step, due to the preference for double coupling, was a drawback of this strategy, enough material could be prepared via this method to characterize and investigate the isomerization properties of the triple photoswitches **3a**/**3b**. Changing the *N*-Boc-*N*-phenylhydrazides used herein to different derivatives opened the possibility to synthesize a wide variety of unsymmetrical starazo species, starting from suitable dibromoazobenzenes.

A preliminary investigation of the presented three-state switches by UV–vis and ^1^H NMR spectroscopy revealed that both derivatives **3a** and **3b** were capable of *E*→*Z* photoisomerization. However, no selective photoswitching could be achieved due to overlapping absorption bands of all arylazobenzene moieties (see Figure 2). Furthermore, analyses showed that starazo compound **3b** had a higher *E*/*Z* ratio in the PSS compared to derivative **3a**. In addition, complex spectra were obtained, in which the individual isomers could not be assigned unambiguously. Although the starazo species that were prepared did not show selective switching, the presented synthesis opened an easy access to other analogous to further study the fundamental properties of 1,3,5-tris(arylazo)benzenes in the near future. These new insights will foster the design of novel, multi-state photoresponsive systems for smart materials.

## Experimental

**3,5-Dibromo-2′,4′,6′-trimethoxyazobenzene (15)** [24]**:** 3,5-Dibromoaniline (9.99 g, 39.8 mmol, 1.00 equiv) was suspended in water (20 mL) and aq HBF_4_ (50%, 15 mL, 119 mmol, 3.0 equiv) was added. The suspension was cooled to 0 °C and a solution of NaNO_2_ (2.76 g, 40.0 mmol, 1.00 equiv) in water (8 mL) was added dropwise. The grey suspension was vigorously stirred at 0 °C for 45 min. The precipitate was filtered off, washed with Et_2_O (5 × 50 mL), and dried in vacuum to yield the diazonium tetrafluoroborate as grey solid (11.2 g). To a suspension of this diazonium tetrafluoroborate (11.2 g, 32.0 mmol, 1.00 equiv) in MeOH (80 mL), a solution of 1,3,5-trimethoxybenzene (5.98 g, 35.6 mmol, 1.11 equiv) in MeOH (80 mL) was added dropwise at rt over 15 min. After standing overnight at −20 °C, the orange precipitate was filtered off, washed with cold MeOH (ca. 25 mL), and dried in vacuum to yield **15** as orange powder (13.8 g, 80%). mp 213–215 °C (dec); ^1^H NMR (400 MHz, DMSO) δ 7.91 (t, *J* = 1.8 Hz, 1H), 7.80 (d, *J* = 1.8 Hz, 2H), 6.38 (s, 2H), 3.89 (s, 3H), 3.83 (s, 6H); ^13^C NMR (101 MHz, DMSO) δ 163.6, 155.6, 155.2, 133.8, 126.2, 123.5, 123.1, 91.5, 56.3, 55.8; HRESIMS (*m*/*z)*: [M + H]^+^ calcd for C_15_H_15_^79^Br_2_N_2_O_3_, 428.9444; found, 428.9448.

***tert-*Butyl (*E*)-2-(3-bromo-5-((2,4,6-trimethoxyphenyl)diazenyl)phenyl)-1-(4-cyanophenyl)hydrazine-1-carboxylate (16a):** In a nitrogen-filled glovebox, an oven-dried Schlenk tube was charged with a 1.61 M solution of P(*t*-Bu)_3_ (395 µL, 636 µmol, 15 mol %) in dry toluene. All following operations were carried out in a fume hood under Schlenk conditions. *tert*-Butyl 1-(4-cyanophenyl)hydrazine-1-carboxylate (1.00 g, 4.25 mmol, 1.00 equiv), **15** (1.82 g, 4.24 mmol, 1.00 equiv), Pd(OAc)_2_ (144 mg, 629 µmol, 15 mol %), and Cs_2_CO_3_ (2.11 mg, 6.40 mmol, 1.51 equiv) were added and suspended in dry toluene (42 mL). After stirring at rt for 35 min, the tube was sealed and heated to 110 °C for 3.5 h. After cooling to rt, the mixture was filtered through a plug of silica gel using EtOAc and concentrated. The mixture was separated by flash column chromatography (SiO_2_, cyclohexane/EtOAc, 2:1, v/v) to yield **16a** as red solid (mixture of isomers, 248 mg, 10%). The product was used without further purification. See Supporting Information File 1 for the ^1^H NMR spectrum. HRESIMS (*m*/*z*): [M + Na]^+^ calcd for C_27_H_28_^79^BrN_5_O_5_Na, 604.1166; found, 604.1167.

***tert*-Butyl (*E*)-2-(3-bromo-5-((2,4,6-trimethoxyphenyl)diazenyl)phenyl)-1-(4-methoxyphenyl)hydrazine-1-carboxylate (16b):** In a nitrogen-filled glovebox, an oven-dried Schlenk tube was charged with P(*t*-Bu)_3_ (155 µL, 628 µmol, 15 mol %). All following operations were carried out in a fume hood under Schlenk conditions. Compound **15** (1.80 g, 4.19 mmol, 1.00 equiv), *tert*-butyl 1-(4-methoxyphenyl)hydrazine-1-carboxylate (1.04 g, 4.36 mmol, 1.04 equiv), Pd(OAc)_2_ (145 mg, 633 µmol, 15.1 mol %), and Cs_2_CO_3_ (2.07 g, 6.29 mmol, 1.5 equiv) were added and suspended in dry toluene (42 mL). The mixture was stirred at rt for 30 min and the tube was sealed and heated to 110 °C for 2 d. After cooling to rt, the reaction mixture was filtered through a plug of silica gel using EtOAc, concentrated, and the residue was separated by flash column chromatography (SiO_2_, cyclohexane/EtOAc, 2:1, v/v) to yield **16b** as red oil (mixture of isomers, 218 mg, 9%). The product was used without further purification. See Supporting Information File 1 for the ^1^H NMR spectrum. HRESIMS (*m*/*z*): [M + H]^+^ calcd for C_27_H_32_^79^BrN_4_O_6_, 587.1500; found, 587.1486.

***ter*t-Butyl (*E*)-2-(3-(2-(*tert*-butoxycarbonyl)-2-(4-cyanophenyl)hydrazinyl)-5-((2,4,6-trimethoxyphenyl)diazenyl)phenyl)-1-phenylhydrazine-1-carboxylate (17a):** In a nitrogen-filled glovebox, an oven-dried Schlenk tube was charged with P(*t*-Bu)_3_ (2.2 µL, 8.9 µmol, 5.2 mol %). The following operations were carried out in a fume hood under Schlenk conditions. To the Schlenk tube, **16a** (100 mg, 172 µmol, 1.00 equiv), *N*-Boc-*N*-phenylhydrazine (110 mg, 528 µmol, 3.08 equiv), Pd(OAc)_2_ (2 mg, 9 µmol, 5 mol %), and Cs_2_CO_3_ (170 mg, 522 µmol, 3.04 equiv) were added. After the addition of dry toluene (1.7 mL), the mixture was stirred at rt for 30 min. Then, the tube was sealed, and the mixture was heated to 110 °C for 2 d. After cooling to rt, the mixture was filtered through a plug of silica gel using EtOAc, and the filtrate was concentrated. The crude mixture was separated by two consecutive flash column chromatography steps (SiO_2_, cyclohexane/EtOAc, 1:1, v/v, then SiO_2_, toluene/EtOAc, 5:1 to 3:1, v/v) to yield **17a** as red oil (mixture of isomers, 28 mg, 23%). The product was used without further purification. See Supporting Information File 1 for the ^1^H NMR spectrum. HRESIMS: [M + H]^+^ calcd for C_38_H_44_N_7_O_7_, 710.3302; found, 710.3427.

***tert*-Butyl (*E*)-2-(3-(2-(*tert*-butoxycarbonyl)-2-(4-methoxyphenyl)hydrazinyl)-5-((2,4,6-trimethoxyphenyl)diazenyl)phenyl)-1-phenylhydrazine-1-carboxylate (17b):** In a nitrogen-filled glovebox, an oven-dried Schlenk tube was charged with P(*t*-Bu)_3_ (9.5 µL, 37 µmol, 15 mol %). All following operations were carried out in a fume hood under Schlenk conditions. To the Schlenk tube, **16b** (150 mg, 255 µmol, 1.00 equiv), *N*-Boc-*N*-phenylhydrazine (164 mg, 787 µmol, 3.08 equiv), Pd(OAc)_2_ (9 mg, 39 µmol, 15 mol %), Cs_2_CO_3_ (255 mg, 783 µmol, 3.07 equiv), and dry toluene (2.5 mL) were added. The mixture was stirred at rt for 30 min. Then, the tube was sealed and the mixture was heated to 110 °C for 24 h. After cooling to rt, the solution was purified by flash column chromatography (SiO_2_, cyclohexane/EtOAc, 2:1, v/v) to yield **17b** as red oil (mixture of isomers, 94 mg, 52%), and the product was used without further purification. See Supporting Information File 1 for the ^1^H NMR spectrum. HRESIMS (*m*/*z*): [M + Na]^+^ calcd for C_38_H_46_N_6_O_8_Na, 737.3269; found, 737.3269.

**(*E*)-1-(4-Cyanophenyl)-2-(3-((*E*)-phenyldiazenyl)-5-((*E*)-(2,4,6-trimethoxyphenyl)diazenyl)phenyl)diazene (3a):** In a dry Schlenk tube under a nitrogen atmosphere, to **17a** (28 mg, 39 µmol, 1.00 equiv) in dry DMF (1 mL) were added CuI (60.0 mg, 313 µmol, 8.0 equiv) and Cs_2_CO_3_ (103 mg, 316 µmol, 8.0 equiv). The tube was sealed and heated to 140 °C for 3 h, cooled to rt, and filtered through a plug of silica gel using EtOAc. The residue was purified by flash column chromatography (SiO_2_, cyclohexane/EtOAc, 2:1, v/v) to yield a red solid (4 mg, 20%), which was recrystallized by slow evaporation from CH_2_Cl_2_ in the dark to yield the all-*E*-isomer **3a**. ^1^H NMR (400 MHz, CDCl_3_) δ 8.56 (t, *J* = 1.9 Hz, 1H), 8.53 (d, *J* = 1.9 Hz, 2H), 8.11–8.03 (m, 2H), 8.03–7.97 (m, 2H), 7.88–7.83 (m, 2H), 7.61–7.50 (m, 3H), 6.27 (s, 2H), 3.94 (s, 6H), 3.92 (s, 3H); ^13^C NMR (101 MHz, CDCl_3_) δ 163.3, 155.9, 155.8, 154.5, 154.3, 154.0, 153.6, 152.6, 149.5, 133.4, 131.8, 129.4, 129.3, 127.7, 123.8, 123.3, 120.0, 119.0, 118.6, 117.7, 114.5, 91.6, 90.7, 56.7, 55.7; HRESIMS (*m*/*z*): [M + H]^+^ calcd for C_28_H_24_N_7_O_3_, 506.1935; found, 506.1935.

**(*E*)-1-(4-Methoxyphenyl)-2-(3-((*E*)-phenyldiazenyl)-5-((*E*)-(2,4,6-trimethoxyphenyl)diazenyl)phenyl)diazene (3b):** A dry Schlenk tube under nitrogen atmosphere was charged with a solution of **17b** (78 mg, 109 µmol, 1.00 equiv), CuI (167 mg, 873 µmol, 8.0 equiv), Cs_2_CO_3_ (284 mg, 872 µmol, 8.0 equiv), and dry DMF (2.8 mL). The tube was sealed and heated to 140 °C for 17.5 h. After cooling to rt, the mixture was filtered through a plug of silica gel using EtOAc, and separated by two consecutive purification steps by flash column chromatography (SiO_2_, cyclohexane/EtOAc, 2:1, v/v) to yield **3b** as red oil (33 mg, 59%). The all-*E*-isomer was obtained by recrystallization through slow evaporation from CHCl_3_ in the dark. ^1^H NMR (400 MHz, CDCl_3_) δ 8.49 (s, 3H), 8.03–7.98 (m, 4H), 7.60–7.47 (m, 3H), 7.04 (d, *J* = 8.9 Hz, 2H), 6.26 (s, 2H), 3.93 (s, 6H), 3.91–3.89 (m, 6H); ^13^C NMR (101 MHz, CDCl_3_) δ 162.9, 162.6, 155.6, 155.3, 155.0, 154.1, 154.0, 153.6, 152.7, 147.1, 131.5, 129.3, 127.8, 125.2, 125.0, 123.2, 118.7, 118.2, 117.3, 116.2, 114.4, 91.6, 56.7, 55.7, 55.7; HRESIMS (*m*/*z*): [M + H]^+^ calcd for C_28_H_27_N_6_O_4_, 511.2089; found, 511.2089.

## Supporting Information

File 1 Additional synthetic procedures, isomerization experiments, and ^1^H/^13^C NMR spectra.
