# Supplementary material for: Starazo triple switches – synthesis of unsymmetrical 1,3,5-tris(arylazo)benzenes
Source: Beilstein J Org Chem. 2020 Jan 3;16:22–31. doi: 10.3762/bjoc.16.4 (PMC6964668; doi:10.3762/bjoc.16.4)
Supplement: File 1 — Additional synthetic procedures, isomerization experiments, and 1H/13C NMR spectra. [file Beilstein_J_Org_Chem-16-22-s001.pdf]

BEILSTEIN JOURNAL OF ORGANIC CHEMISTRY

Supporting Information

for

Starazo triple switches – synthesis of unsymmetrical 1,3,5-tris(arylazo)benzenes

Andreas H. Heindl and Hermann A. Wegner

Beilstein J. Org. Chem. **2020,** *16,* 22–31. doi:10.3762/bjoc.16.4

Additional synthetic procedures, isomerization experiments, and $^{1}H/^{13}C$ NMR spectra

License and Terms: This is a supporting information file under the terms of the Creative Commons Attribution License (https://creativecommons.org/licenses/by/4.0). Please note that the reuse, redistribution and reproduction in particular requires that the authors and source are credited.
The license is subject to the *Beilstein Journal of Organic Chemistry* terms and conditions: (https://www.beilstein-journals.org/bjoc)

General information S1

Synthesis... S3

^{1}H NMR isomerization experiments S7

^{1}H and ^{13}C{^{1}H} NMR spectra S9

References S18

General information

Chemicals were used as purchased from Sigma-Aldrich, Acros Organics, Alfa Aesar, and TCI Europe. Anhydrous solvents were purchased from Acros Organics. Technical grade solvents used during workup and purification were distilled prior to use. Air- and/or moisture-sensitive reactions were carried out under Schlenk conditions or in a nitrogen-filled glovebox. Solids were dried under high vacuum (oil pump, ca. 10^{-3} mbar) at rt, 50 °C, or 60 °C, as necessary. Flash column chromatography and column chromatography were carried out using Silica 60 M (0.04–0.063 mm) from Macherey-Nagel. Thin-layer chromatography was performed on Polygram® SIL G/UV_{254} from Macherey-Nagel. NMR spectra were recorded on a Bruker Avance II 200 MHz, Avance II 400 MHz, or Avance III 400 MHz HD spectrometer at rt, if not stated otherwise. Chemical shifts are reported in ppm relative to the solvent peak, coupling constants *J* are reported in Hz. Deuterated solvents were obtained from Deutero GmbH or Eurisotop. For all azobenzenes, the thermodynamically more stable *E*-isomer is reported, if not noted otherwise. HRESIMS spectra were recorded on a Bruker Daltonics Micro TOF. Melting points were measured on a Krüss M5000 capillary melting point meter with a heating rate of 1 °C/min. Solvents for UV–vis spectroscopy were purchased from Merck (Uvasol® quality). The UV–vis measurements were carried out with a SPECORD® 200 PLUS spectrophotometer equipped with two automatic eight-fold cell changers and a Peltier element thermostat system (0.1 °C accuracy) by Analytik Jena. The system was operated with the ASpect UV software by Analytik Jena. The sample solutions were measured in QS High-precision Cells made of Quartz Suprasil® with a light path of 10 mm by Hellma Analytics.

Irradiation experiments

Irradiation of the NMR or UV–vis samples was conducted in an in-house-built box using high-power LEDs by Lumitronix or Nichia. After the given irradiation time, the samples were immediately placed and measured in the corresponding spectrometer.

Table S1: LEDs used for all irradiation experiments and their specifications.

λ_{max} (nm)	$\Delta\lambda_{FWHM}$ (nm)	luminous flux (mW)	product name
365	9	780	NCSU276AT-U365
448	20	520	LXML-PR01-0500

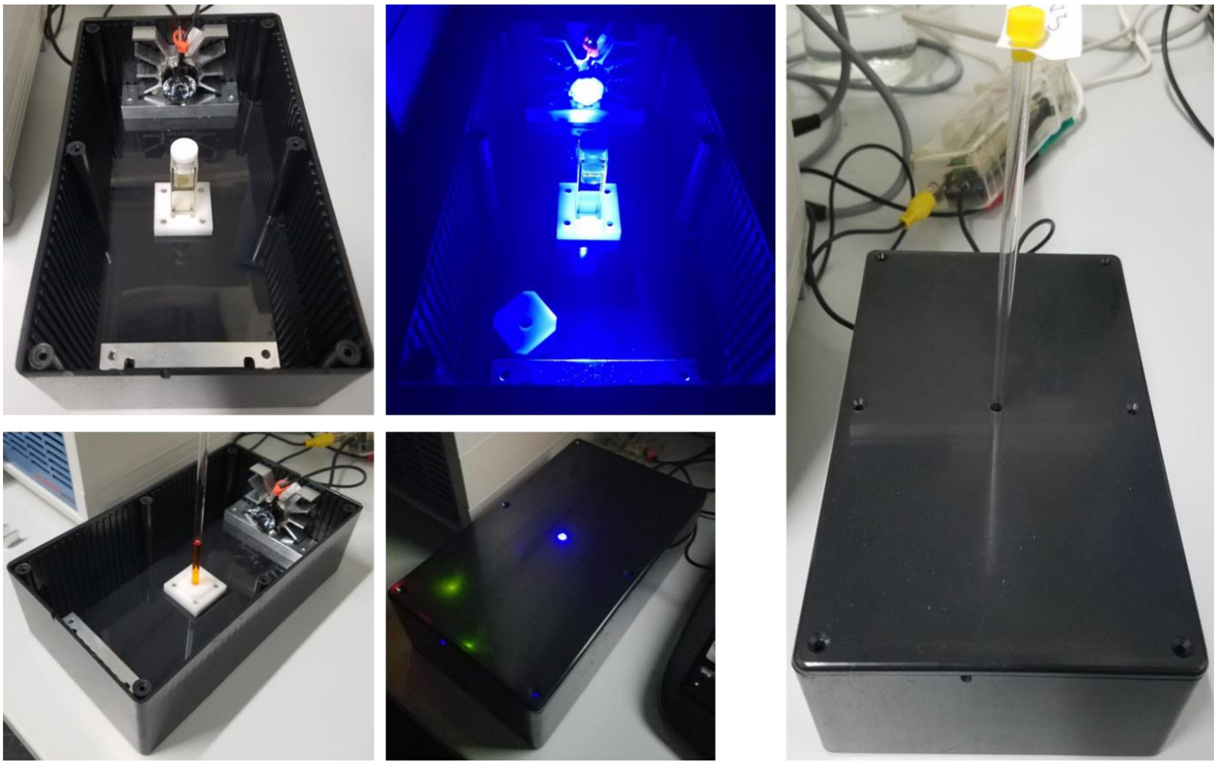

Figure S1: In-house-built LED irradiation box for UV–vis cuvettes as well as NMR tubes.

Synthesis

***tert*-Butyl 1-(4-methoxyphenyl)hydrazine-1-carboxylate** [1]:

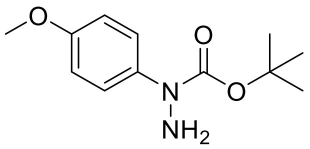

4-Iodoanisole (4.02 g, 16.8 mmol, 1.00 equiv), *tert*-butyl carbazate (2.70 g, 20.2 mmol, 1.20 equiv), CuI (3.14 g, 16.4 mmol, 0.975 equiv), and Cs_2CO_3 (8.25 g, 25.1 mmol, 1.49 equiv) were suspended in DMSO (15 mL), and the mixture was stirred at 50 °C for 40 min. Afterwards, the reaction mixture was diluted with EtOAc, filtered through a plug of silica gel (EtOAc wash), and the concentrated filtrate was purified by flash chromatography (SiO_2, cyclohexane/EtOAc, 2:1, v/v) to yield the product as pale yellow oil (2.46 g, 61%). ^{1}H NMR (400 MHz, $CDCl_3$) δ 7.30 (d, *J* = 9.0 Hz, 2H), 6.84 (d, *J* = 9.0 Hz, 2H), 4.44 (s, 2H), 3.79 (s, 3H), 1.47 (s, 9H). Analytical data corresponded to the literature [1].

***tert*-Butyl 1-(4-cyanophenyl)hydrazine-1-carboxylate** [2]:

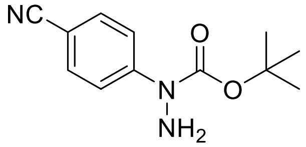

An oven-dried Schlenk tube under a nitrogen atmosphere was charged with 4-bromobenzonitrile (1.85 g, 10.1 mmol, 1.00 equiv), *tert*-butyl carbazate (2.70 g, 20.0 mmol, 1.99 equiv), Cs_2CO_3 (3.27 g, 10.0 mmol, 0.997 equiv), and Pd(dppf)Cl_2·CH_2Cl_2 (85 mg, 0.10 mmol, 1.0 mol %) as well as dry toluene (20 mL). The tube was sealed and the suspension was stirred at 100 °C for 18.5 h. After cooling to rt, the mixture was filtered through a plug of silica gel (EtOAc wash), and concentrated to yield a brown oil, which was purified by flash column chromatography (SiO_2, cyclohexane/EtOAc, 3:1, v/v) to yield an off-white solid (1.83 g, 78%). ^{1}H NMR (400 MHz, $CDCl_3$) δ 7.75 (d, *J* = 8.9 Hz, 1H), 7.57 (d, *J* = 8.9 Hz, 1H), 4.38 (s, 2H), 1.55 (s, 9H). m.p. 107 °C. Analytical data corresponded to the literature [3].

***tert*-Butyl 1-phenylhydrazine-1-carboxylate** [1]:

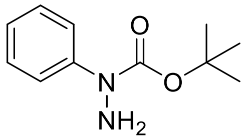

Iodobenzene (5.02 g, 24.6 mmol, 1.00 equiv), *tert*-butyl carbazate (3.96 g, 29.4 mmol, 1.19 equiv), CuI (237 mg, 1.24 mmol, 5.03 mol %), and Cs_2CO_3 (12.2 g, 37.0 mmol, 1.51 equiv) were suspended in DMSO (20 mL), and the mixture was stirred at 50 °C for 2.5 h. The mixture was filtered through a plug of silica gel (EtOAc wash), and the solvents were evaporated under reduced pressure. The crude product was purified by flash column chromatography (SiO_2, cyclohexane/EtOAc, 5:1, v/v) to yield the product as a yellow oil (3.45 g, 67%). ^{1}H NMR (400 MHz, $CDCl_3$) δ 7.48 – 7.43 (m,

2H), 7.34 – 7.27 (m, 2H), 7.14 – 7.08 (m, 1H), 4.44 (s, 2H), 1.50 (s, 9H). Analytical data corresponded to the literature [3].

***N*-(3,5-Dinitrophenyl)acetamide (5):**

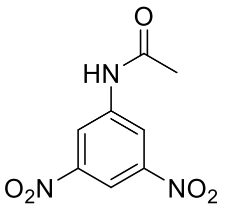

A suspension of 3,5-dinitrophenylaniline (15.9 g, 86.7 mmol, 1.00 equiv) and Ac_2O (17.0 ml, 179 mmol, 2.10 equiv) in AcOH (40 mL) was stirred for a few minutes during which it warmed up and turned pale yellow. When the reaction mixture had cooled back to rt, the precipitate was filtered off, washed with water, and dried in vacuum to yield the product as yellow solid (18.5 g, 95 %). ^{1}H NMR (400 MHz, DMSO-d_6) δ 10.83 (s, 1H), 8.80 (d, J = 2.1 Hz, 2H), 8.47 (t, J = 2.1 Hz, 1H), 2.14 (s, 3H). ^{13}C NMR (101 MHz, DMSO-d_6) δ 169.7, 148.2, 141.2, 118.1, 112.0, 24.1. HRMS (ESI): *m/z* for $C_8H_7N_3O_5Na^{++}$; calcd. 248.0278, found 248.0283. m.p. 191 °C.

***N*-(3-Amino-5-nitrophenyl)acetamide (6)**[4]**:**

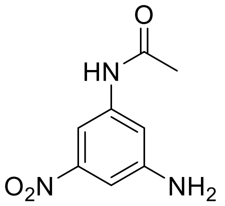

To a refluxing solution of **5** (10.0 g, 44.4 mmol, 1.00 equiv) in EtOH (270 mL), a solution of aqueous $(NH_4)_2S$ (20%, 46.0 mL, 135 mmol, 3.00 equiv) in EtOH (22 mL) was added dropwise over 75 min. The reaction mixture was refluxed for further 30 min and was allowed to cool to rt afterwards. The yellow precipitate formed was filtered off, and the filtrate was concentrated under reduced pressure. The residue was treated with water (220 mL) and conc HCl (27 mL) and was filtered. The filtrate was neutralized with aq NaOH (10%, ca. 140 mL), and the precipitate formed was filtered off, washed with H_2O, and recrystallized from H_2O/EtOH, 10:1, v/v. The obtained orange needles were filtered off, washed with water, and dried in vacuum. The product was obtained as orange needles (5.67 g, 65%). ^{1}H NMR (200 MHz, DMSO-d_6) δ 10.07 (s, 1H), 7.63 (t, J = 2.0 Hz, 1H), 7.22 (t, J = 2.0 Hz, 1H), 7.07 (t, J = 2.2 Hz, 1H), 5.83 (s, 2H), 2.04 (s, 3H). m.p. 203 °C (decomposition). Analytical data corresponded to the literature [4].

***N*-(3-Nitroso-5-nitrophenyl)acetamide (7)** [5]**:**

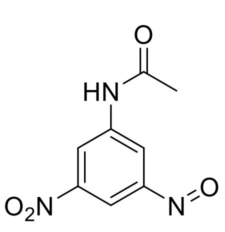

6 (1.00 g, 5.12 mmol, 1.0 equiv) was suspended in DCM (90 mL), and a solution of Oxone® (6.31 g, 10.3 mmol, 4.00 equiv) in water (180 mL) was added. The mixture was stirred vigorously for 30 min and EtOAc (ca. 500 mL) was added afterwards. After phase separation, the aq phase was

extracted with EtOAc (170 mL), and the combined organic phases were dried over $MgSO_4$, filtered, and concentrated to yield a green/brown solid (1.06 g, 99%). The product was used without further purification.

3-Acetamido-5-nitroazobenzene (8):

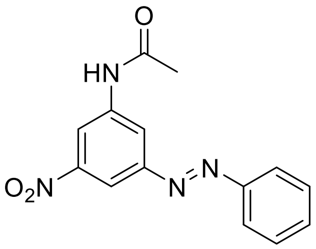

A suspension of **7** (142 mg, 680 µmol, 1.50 equiv) in AcOH (1 mL) was degassed with a nitrogen stream for 5 min, and freshly distilled aniline (42 µL, 0.46 mmol, 1.00 equiv) was added. The reaction mixture was stirred at rt under a nitrogen atmosphere for ca 24 h and water (5 mL) was added afterwards. Following this, the brown precipitate was filtered and washed with water. The crude product was dissolved in hot EtOH, cooled, and the brown impurity was filtered off. The red filtrate was concentrated under reduced pressure to yield a brown solid (114 mg, 87%). Alternatively, the crude product could be purified by column chromatography (SiO_2, toluene/EtOAc; 1:1). ^{1}H NMR (400 MHz, DMSO-d_6) δ 10.67 (s, 1H), 8.70 (t, J = 2.1 Hz, 1H), 8.47 (t, J = 1.8 Hz, 1H), 8.25 (t, J = 2.0 Hz, 1H), 8.02 – 7.89 (m, 2H), 7.74 – 7.54 (m, 3H), 2.14 (s, 3H). ^{13}C NMR (101 MHz, DMSO-d_6) δ 169.3, 152.3, 151.5, 148.8, 141.2, 132.6, 129.6, 123.0, 118.3, 114.8, 110.7, 24.1. HRMS (ESI): m/z for $C_{14}H_{12}N_4O_3Na^+$; calcd. 307.0801, found 307.0800. m.p. 193 °C.

3,5-Dibromoazobenzene (11) [6]**:**

To a solution of 3,5-dibromoaniline (2.01 g, 8.01 mmol, 1.00 equiv) in AcOH (10 mL),

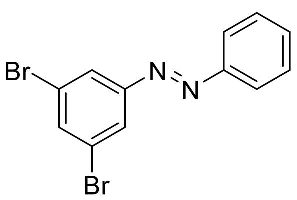

nitrosobenzene (1.07 g, 9.69 mmol, 1.21 equiv) was added, and the mixture was stirred at rt for 25 h. The precipitate was filtered off and consecutively washed with AcOH and water, and was dried in vacuum to yield a brown solid (1.83 g, 67%). ^{1}H NMR (400 MHz, $CDCl_3$) δ 8.01 (d, J = 1.8 Hz, 2H), 7.95 – 7.89 (m, 2H), 7.76 (t, J = 1.8 Hz, 1H), 7.59 – 7.49 (m, 3H). ^{13}C NMR (101 MHz, $CDCl_3$) δ 154.1, 152.3, 135.9, 132.2, 129.4, 125.0, 123.6, 123.4. HRMS (ESI): m/z for $[M+H]^+$; calcd. 338.9127, found 338.9132. m.p. 110 – 114 °C (decomposition). Analytical data corresponded to the literature [6].

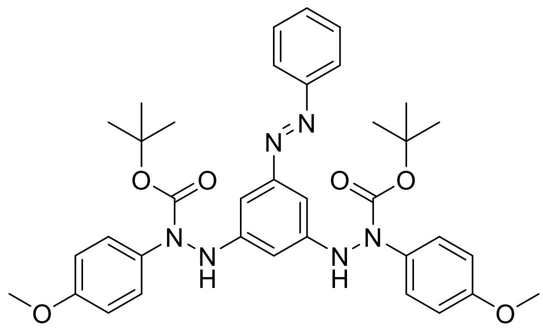

3,5-Bis(4-methoxyphenylhydrazo)azobenzene 13:

In a nitrogen-filled glovebox, an oven-dried Schlenk tube was charged with P(*t*-Bu)$_3$ (5 µL, 20 µmol, 5 mol %) and *tert*-butyl-1-(4-methoxyphenyl)hydrazine-1-carboxylate (209 mg, 877 µmol, 2.18 equiv) in dry toluene (5 mL). The following operations were carried out in a fume hood under Schlenk-conditions. **11** (137 mg, 403 µmol, 1.00 equiv), Cs_2CO_3 (200 mg, 608 µmol, 1.51 equiv), and Pd(OAc)$_2$ (5 mg, 22 µmol, 5 mol %) were added. The mixture was stirred at rt for 30 min and the tube was sealed and heated to 110 °C for 2 h and 15 min afterwards. The cooled mixture was filtered through a plug of silica gel (EtOAc wash), and the concentrated filtrate was purified by flash column chromatography (SiO_2, cyclohexane/EtOAc, 2:1, v/v) to yield a yellow solid (152 mg, 58%). ^{1}H NMR (400 MHz, $CDCl_3$) δ 7.91 – 7.77 (m, 2H), 7.53 – 7.38 (m, 7H), 6.99 (d, J = 2.1 Hz, 2H), 6.94 – 6.70 (m, 4H), 6.48 (s, 2H), 6.41 (t, J = 2.1 Hz, 1H), 3.79 (s, 6H), 1.37 (s, 18H). ^{13}C NMR (101 MHz, $CDCl_3$) δ 157.0, 154.6, 154.3, 152.7, 150.2, 136.0, 131.1, 129.2, 124.0, 123.0, 113.9, 101.0, 100.0, 82.3, 55.6, 28.3. HRMS (ESI): *m*/*z* for $C_{36}H_{42}N_6O_6Na^+$; calcd. 677.3058, found 677.3059. m.p. 93 °C.

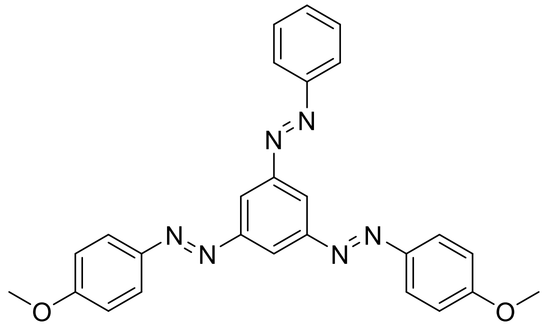

Bis-3,5-(4-methoxyphenylazo)azobenzene (14):

An oven-dried Schlenk-tube under a N_2-atmosphere was charged with **13** (46 mg, 70 µmol, 1.0 equiv), CuI (55 mg, 29 µmol, 4.1 equiv), Cs_2CO_3 (95 mg, 29 µmol, 4.2 equiv), and dry DMF (1 mL). The tube was sealed, and the mixture was heated to 140 °C for 3 d. After cooling to rt, the mixture was filtered through a plug of silica gel (EtOAc wash), concentrated, and the residue was purified by flash column chromatography (SiO_2, toluene/cyclohexane, 1:1 to 1:0, v/v) to yield the product as orange solid (6 mg, 19%). ^{1}H NMR (400 MHz, $CDCl_3$) δ 8.51 (s, 3H), 8.07 – 7.97 (m, 6H), 7.61 – 7.48 (m, 3H), 7.05 (d, J = 9.0 Hz, 4H), 3.91 (s, 6H). ^{13}C NMR (101 MHz, $CDCl_3$) δ 162.7, 154.2, 154.0, 152.7, 147.0, 131.6, 129.3, 125.3, 123.3, 118.6, 118.2, 114.5, 55.8. HRMS (ESI): *m*/*z* for $C_{26}H_{22}N_6O_2Na^+$; calcd. 473.1696, found 473.1693.

^{1}H NMR isomerization experiments

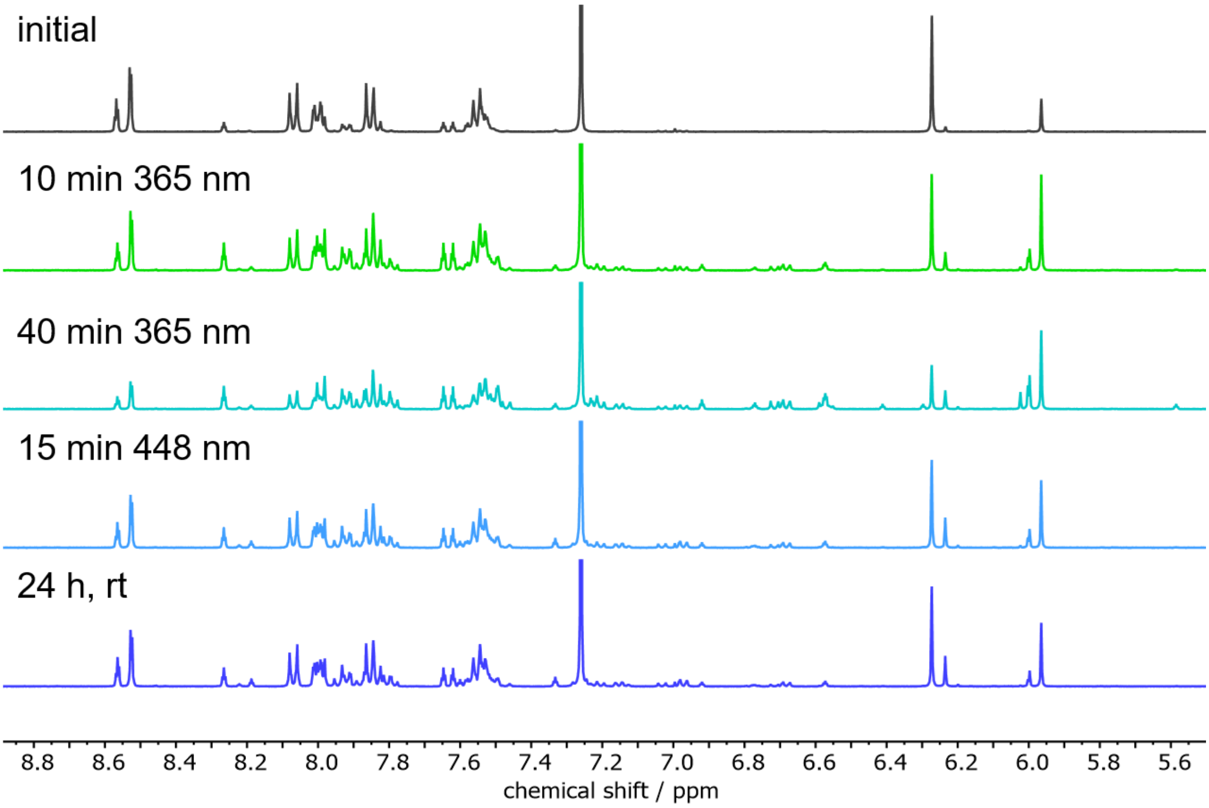

Figure S2: ^{1}H NMR spectra taken during the photoisomerization process of starazo compound **3a**. Upon irradiation at 365 nm, isomerization to multiple photoisomers could be observed, which could not be unambiguously assigned. When irradiating at 448 nm, the initial spectrum could be roughly restored (light blue spectrum). After standing at rt in the dark overnight, slow thermal back-isomerization could be observed (blue spectrum).

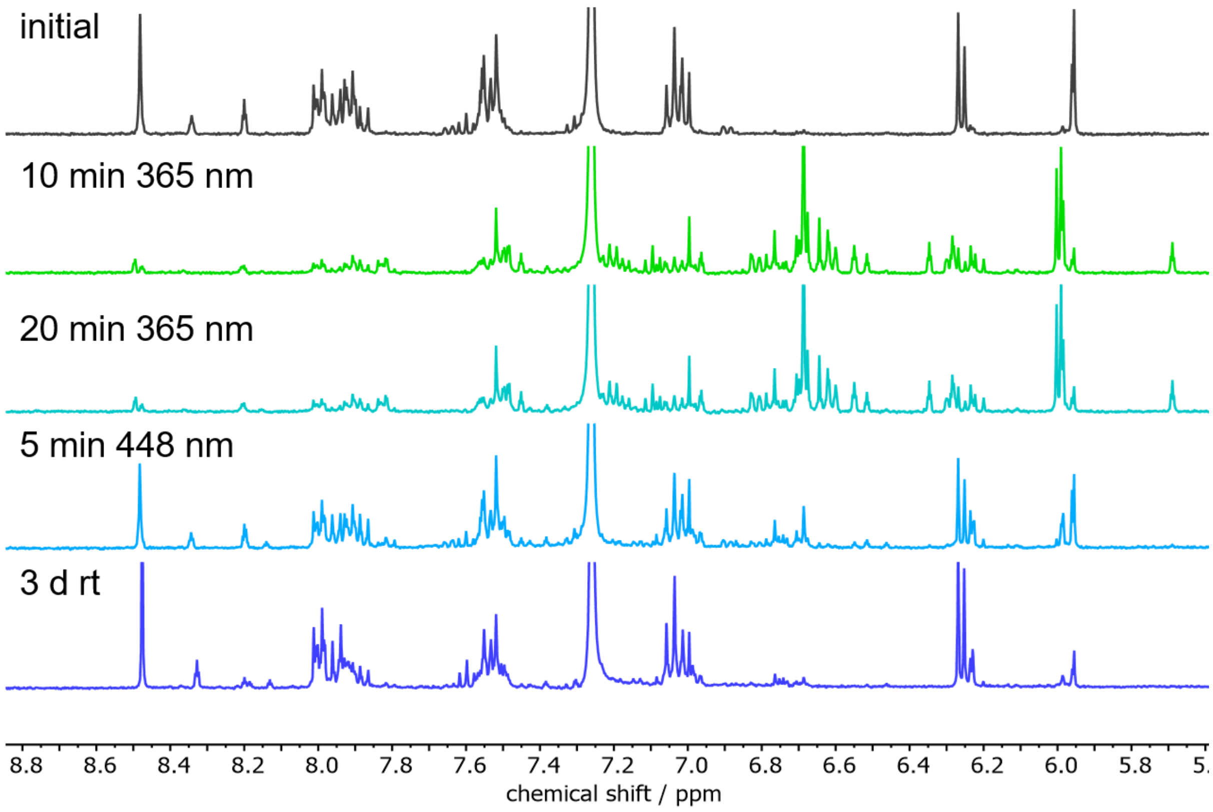

Figure S3: ^{1}H NMR spectra taken during the photoisomerization process of starazo species **3b**. Upon irradiation at 365 nm, complex spectra of multiple isomerization states were obtained (green and cyan spectra), which could not be assigned unambiguously. When irradiating at 448 nm, the initial spectrum could be roughly restored (light blue spectrum). After standing at rt in the dark for 3 d, the initial spectrum was almost restored (blue spectrum).

^{1}H and $^{13}C\{^{1}H\}$-NMR spectra

N-(3,5-Dinitrophenyl)acetamide (5)

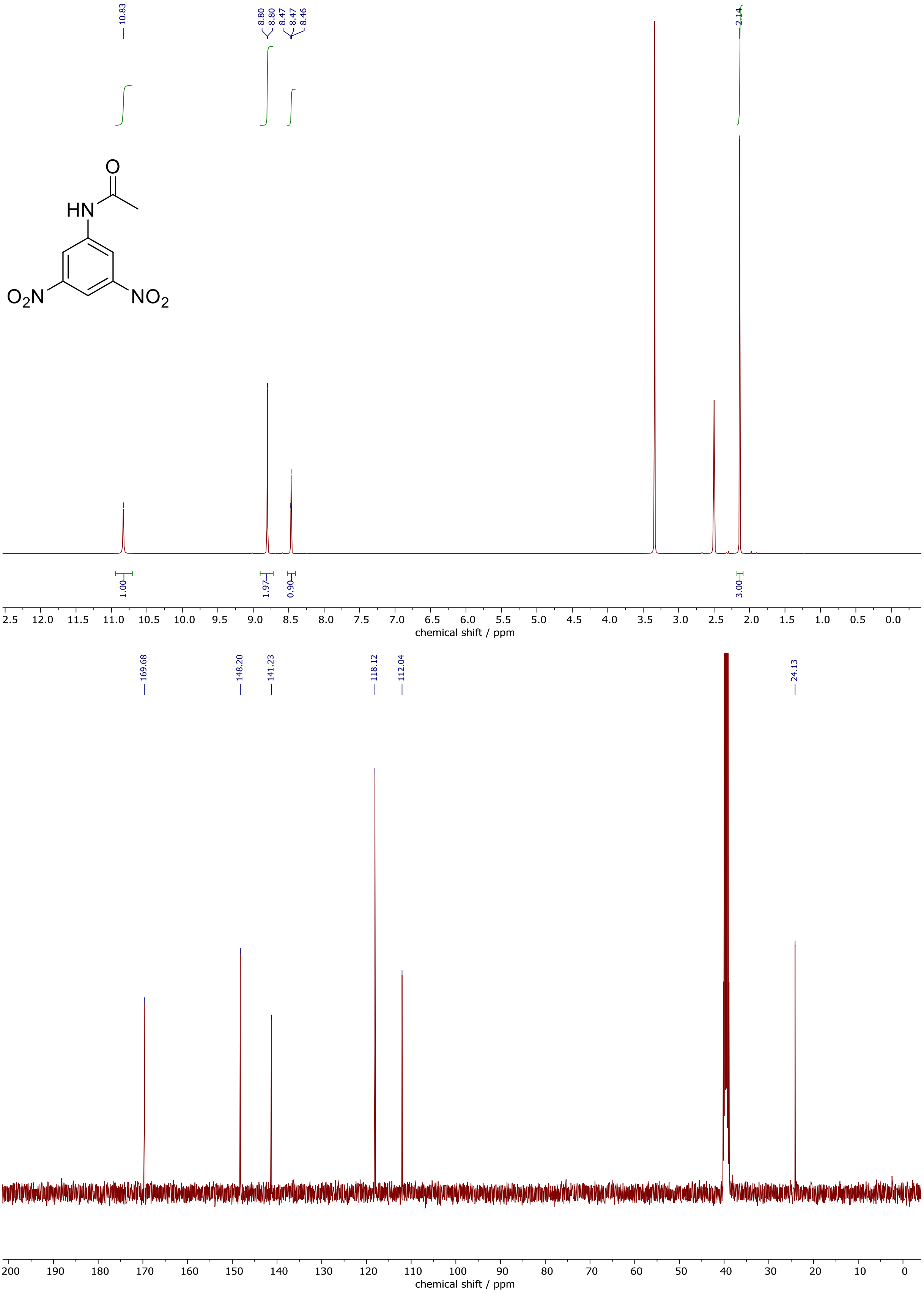

3-Acetamido-5-nitroazobenzene (8)

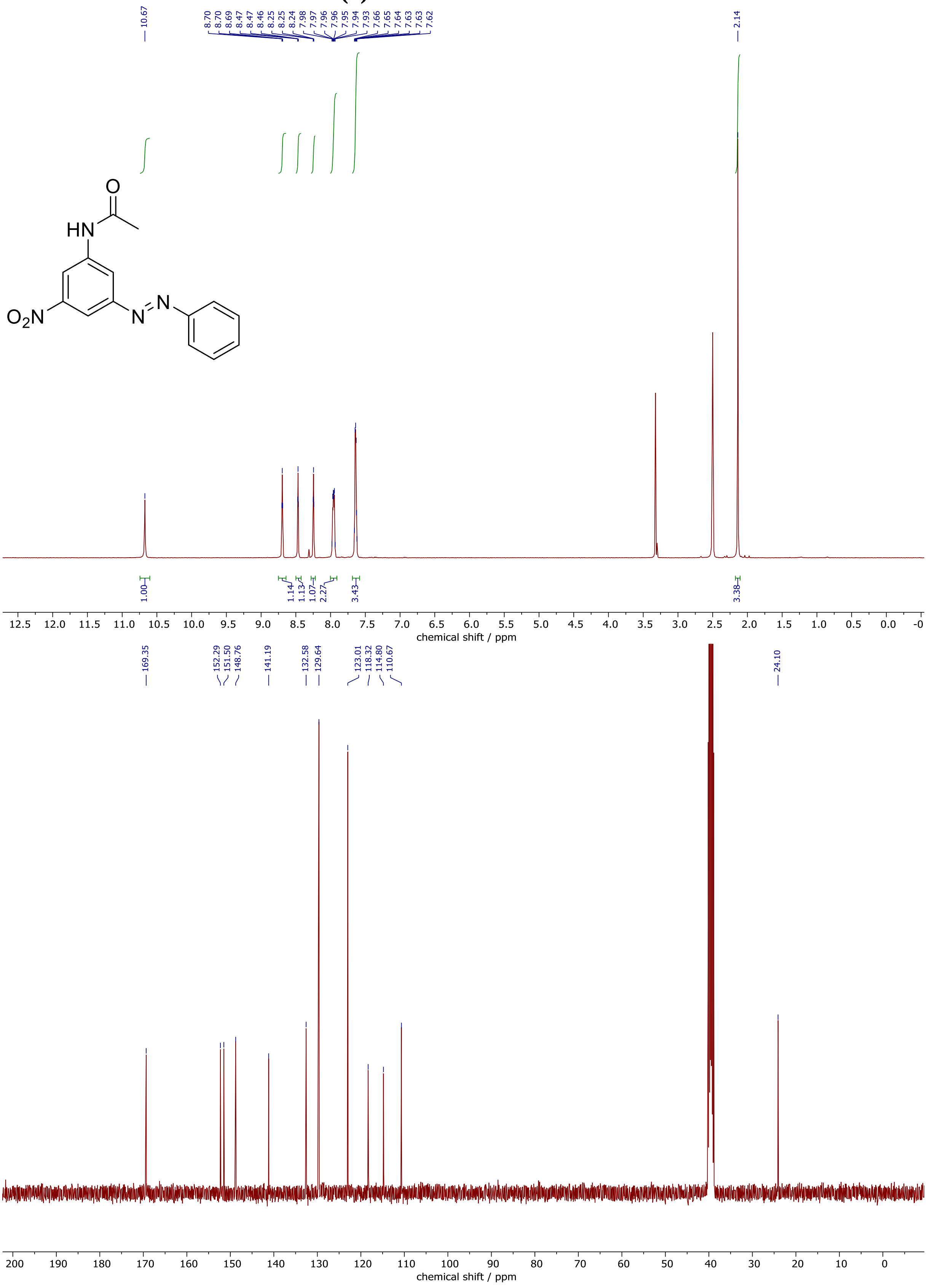

3,5-Bis(4-methoxyphenylhydrazo)azobenzene 13

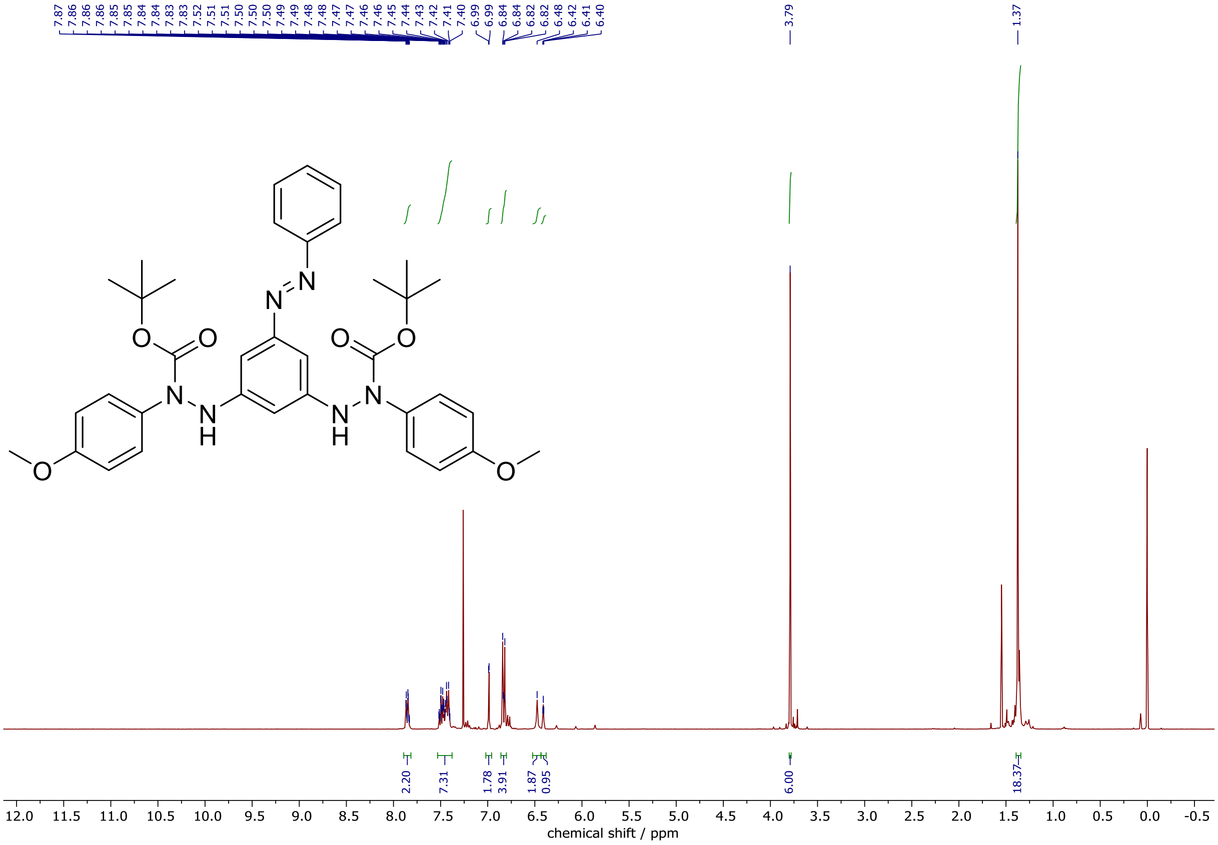

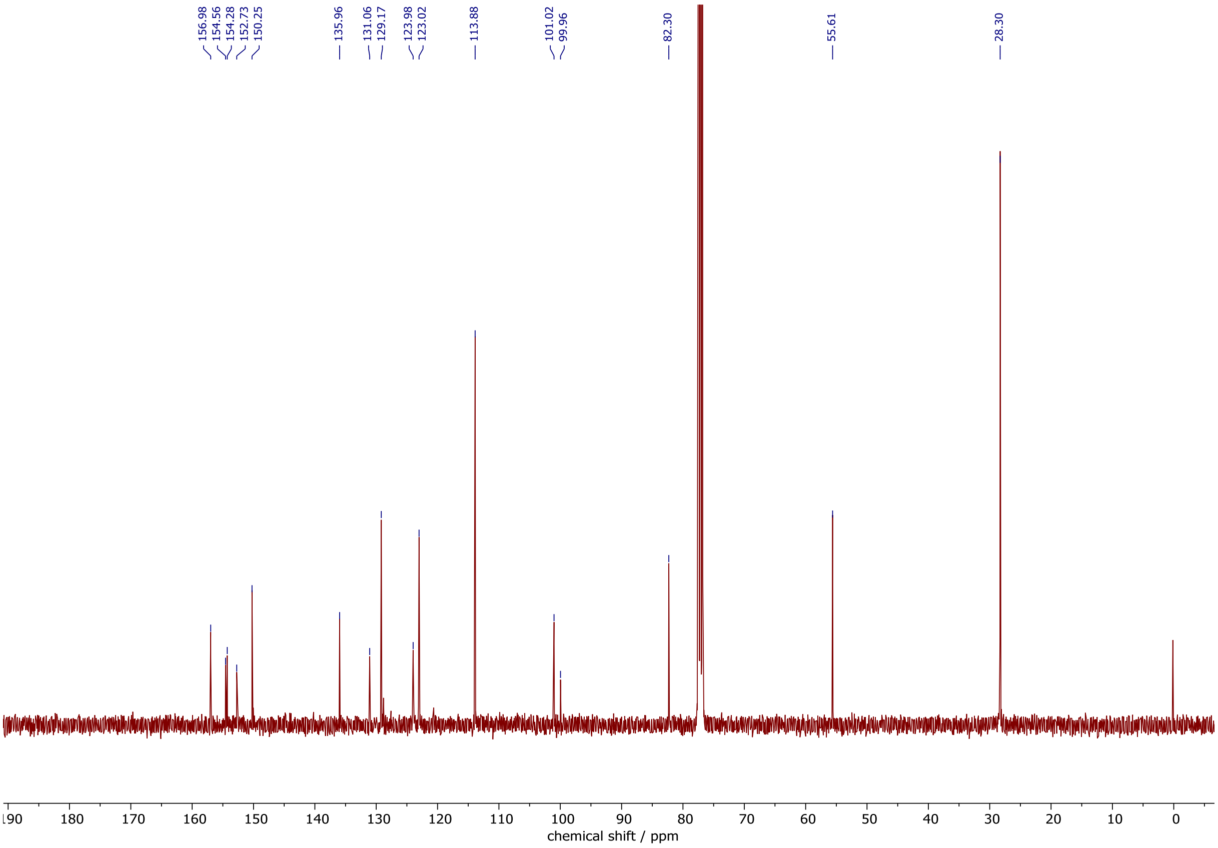

Bis-3,5-(4-methoxyphenylazo)azobenzene (14)

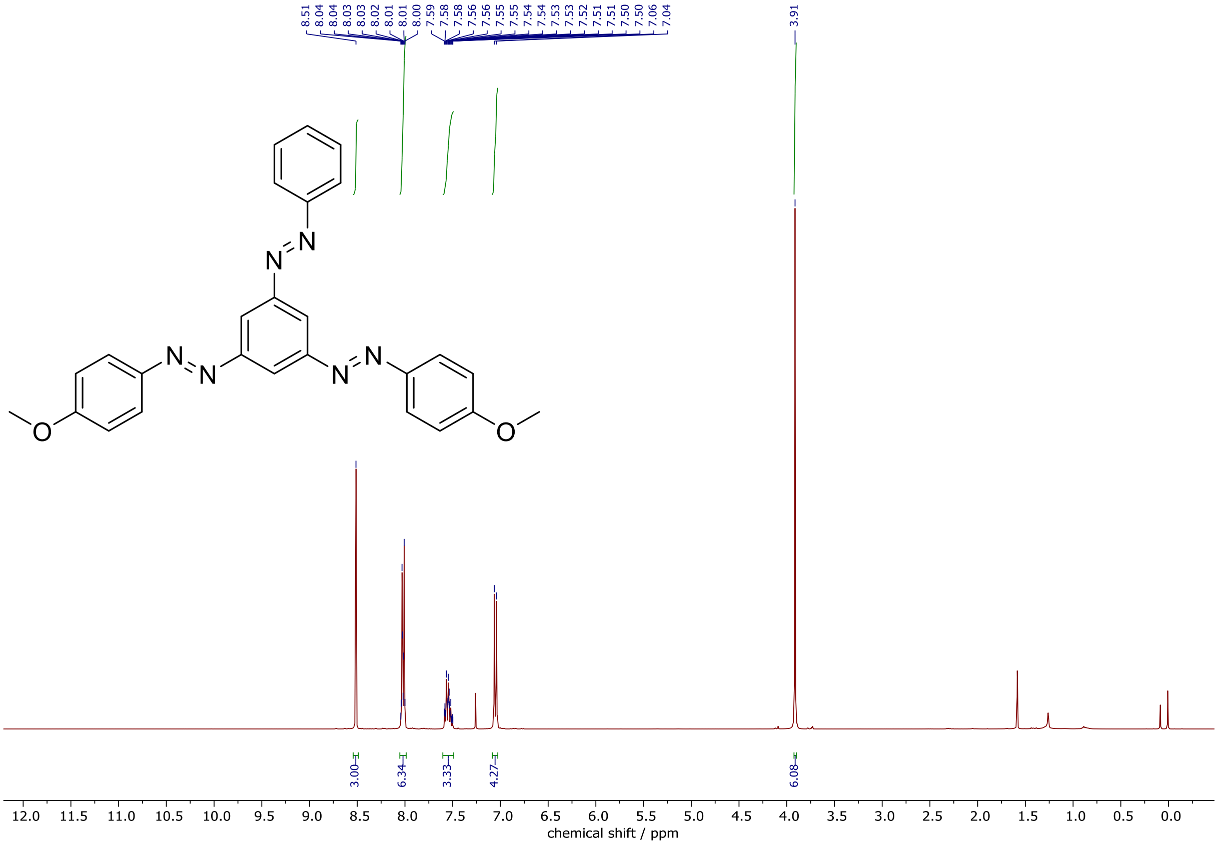

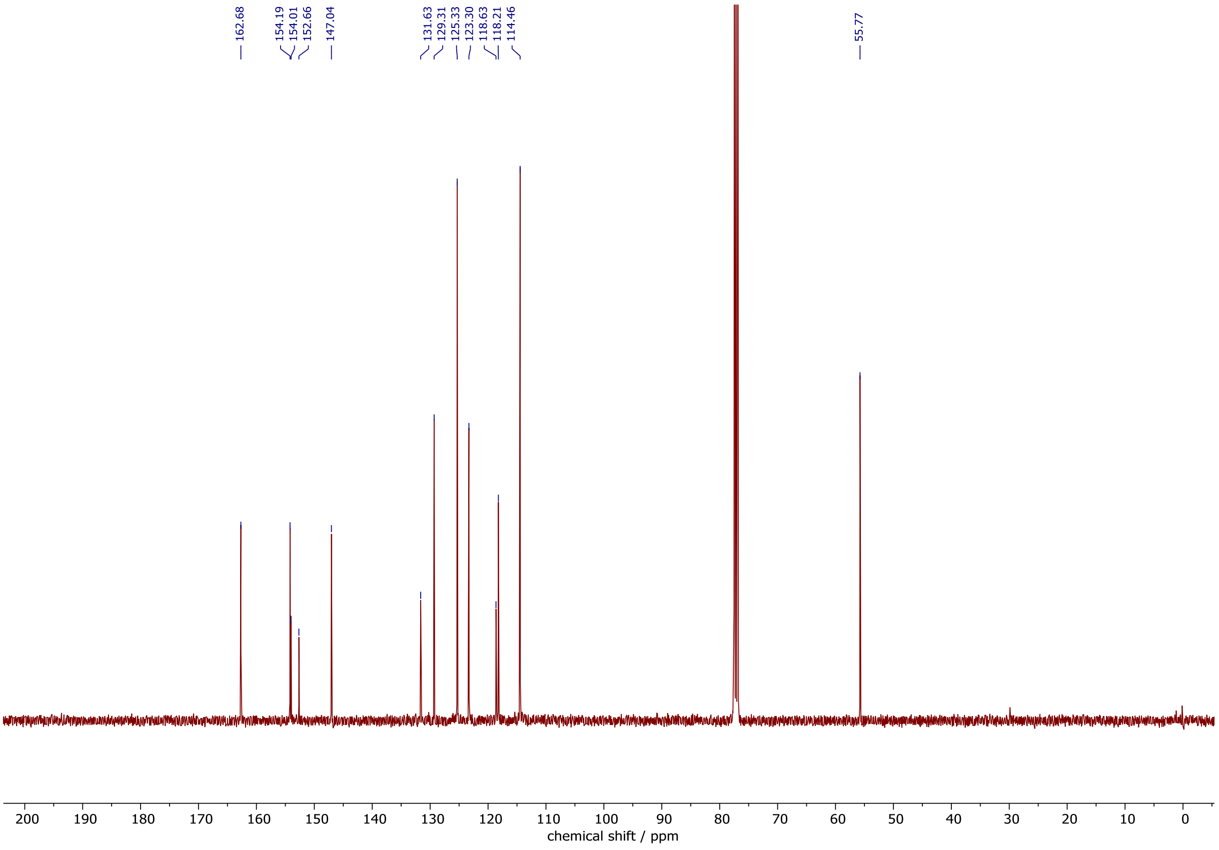

3,5-Dibromo-2’,4’,6’-trimethoxyazobenzene (15)

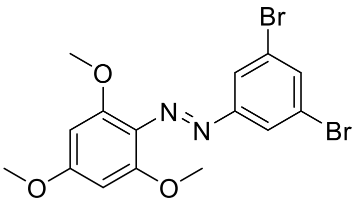

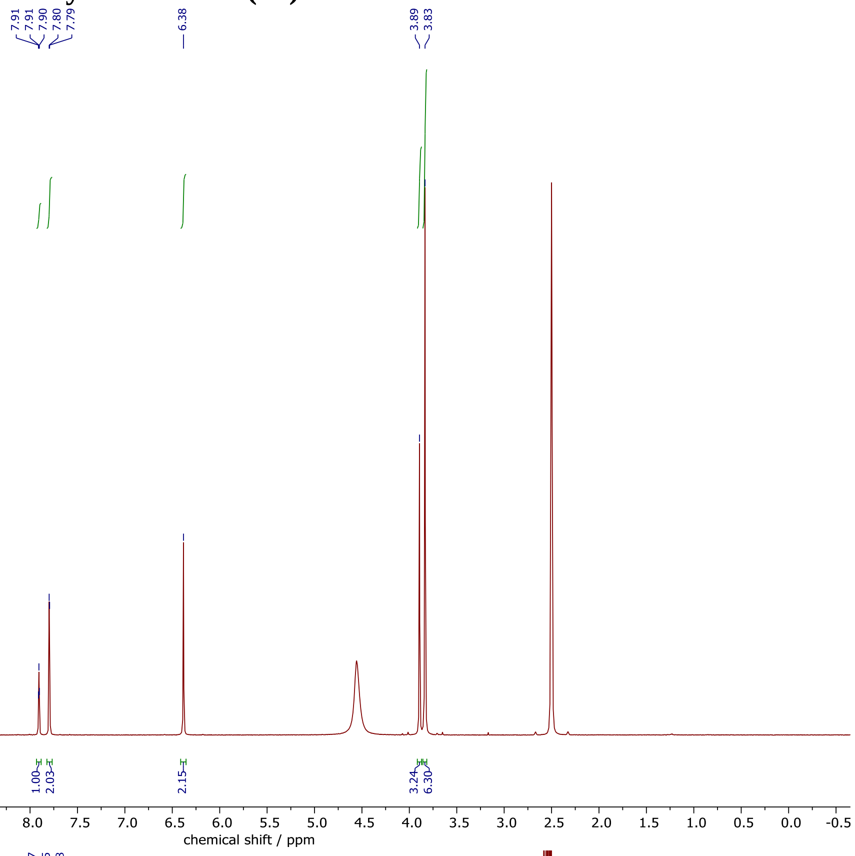

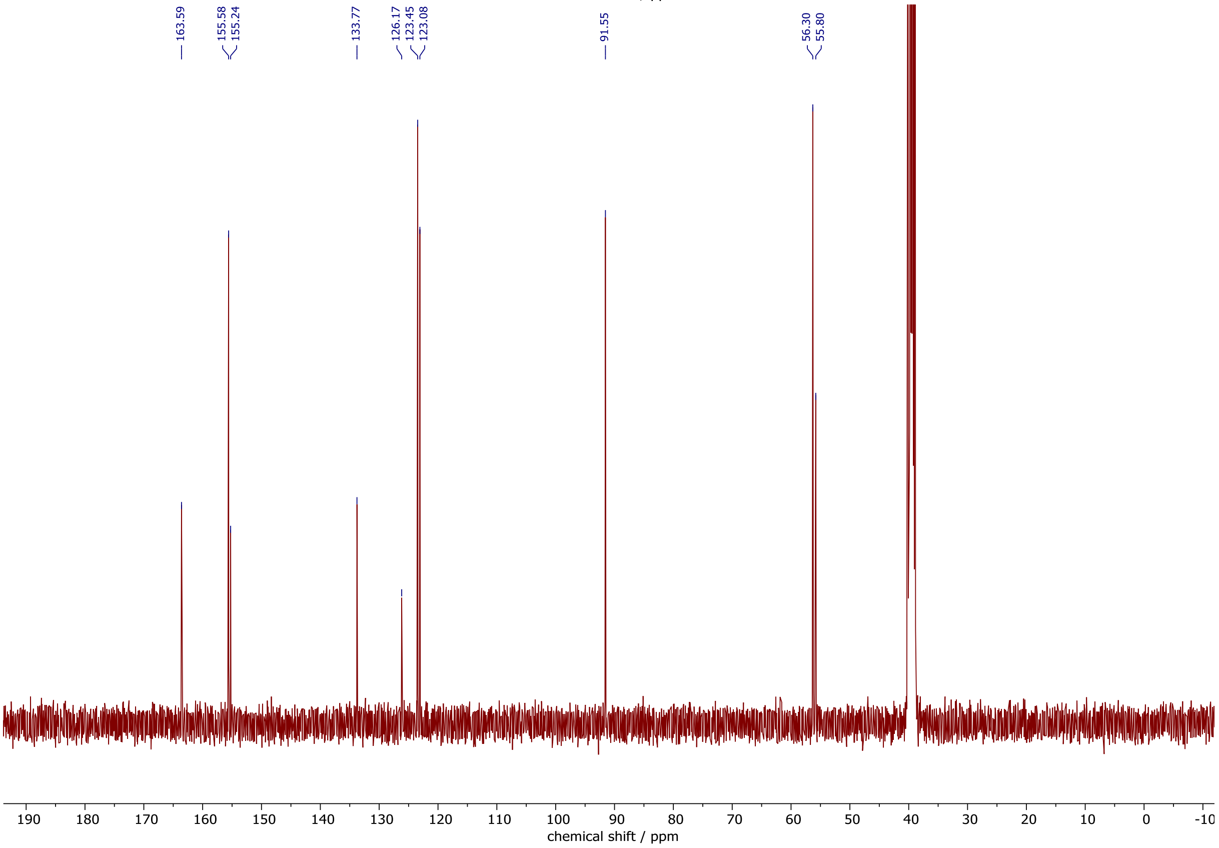

Azo-*N*-Boc-arylhydrazide 16a

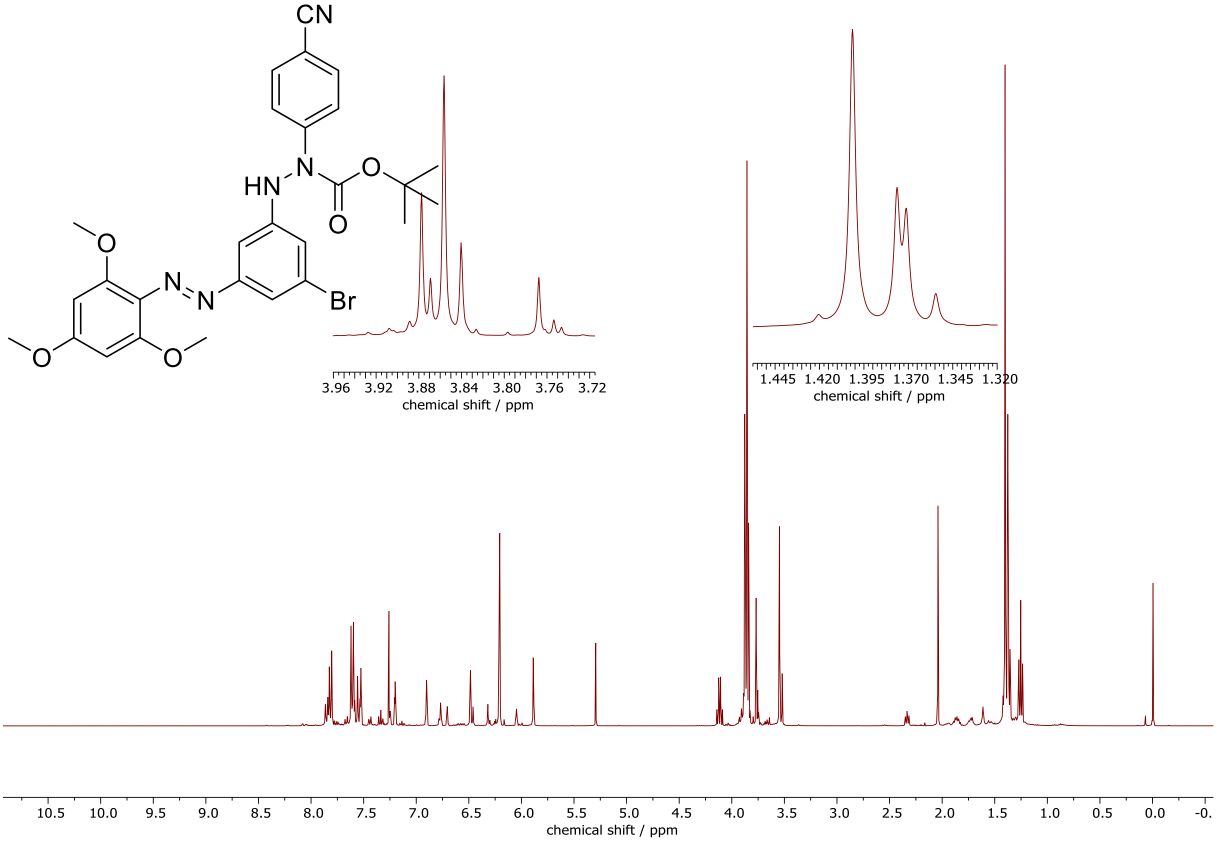

Azo-*N*-Boc-arylhydrazide 16b

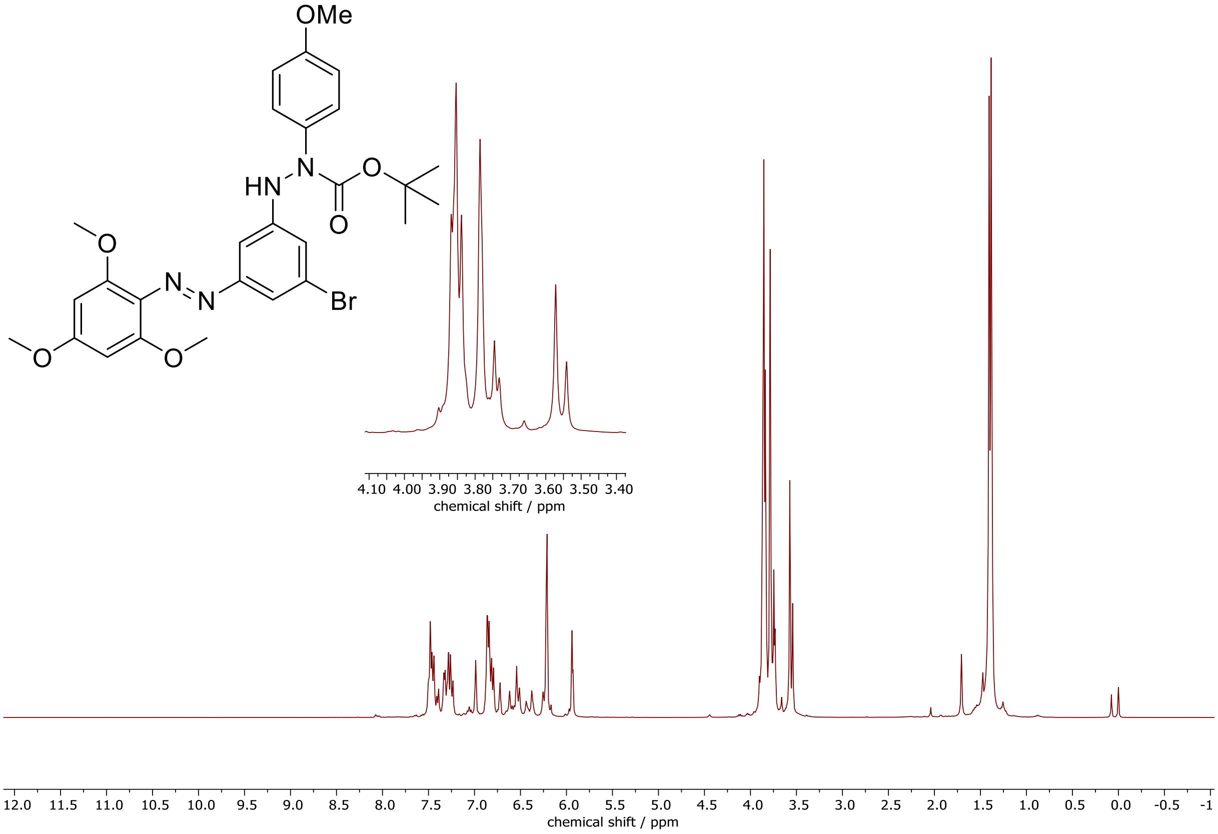

Azo-bis-*N*-Boc-arylhydrazide 17a

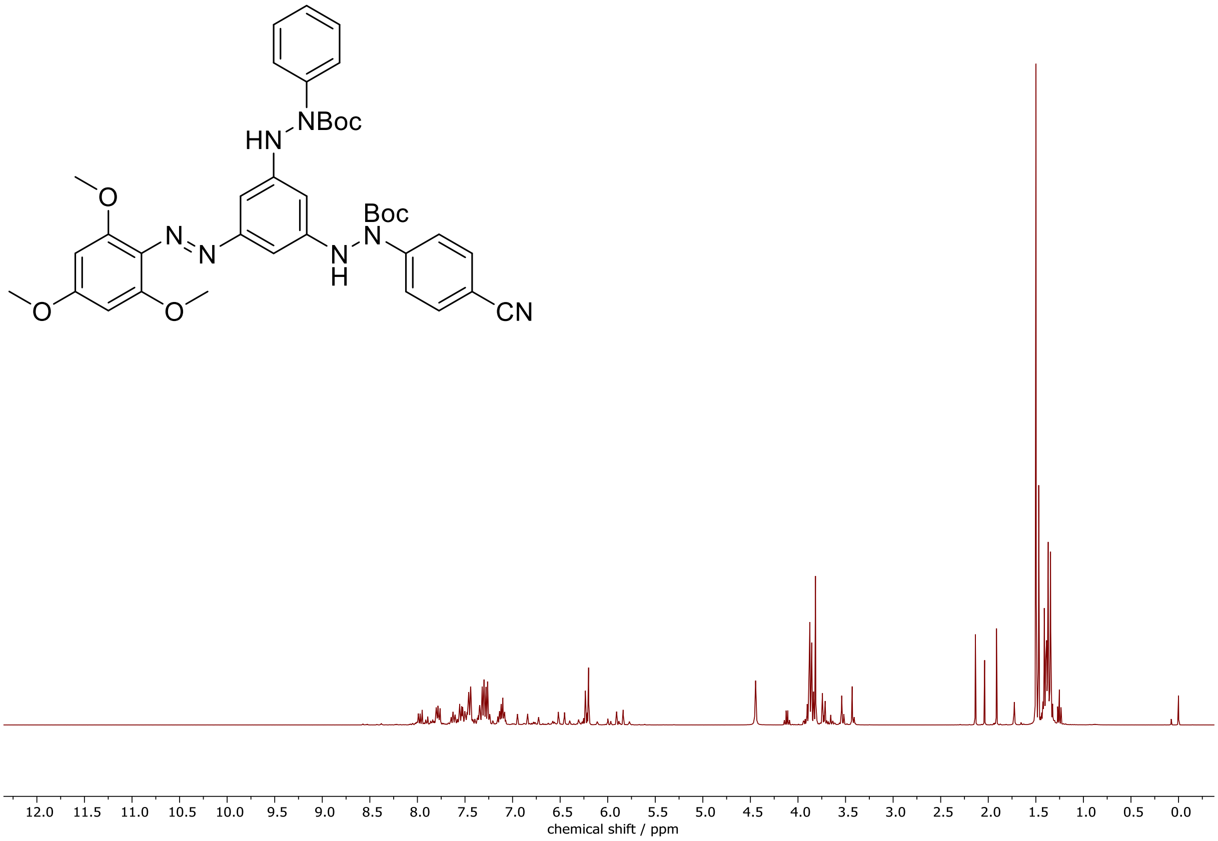

Azo-bis-*N*-Boc-arylhydrazide 17b

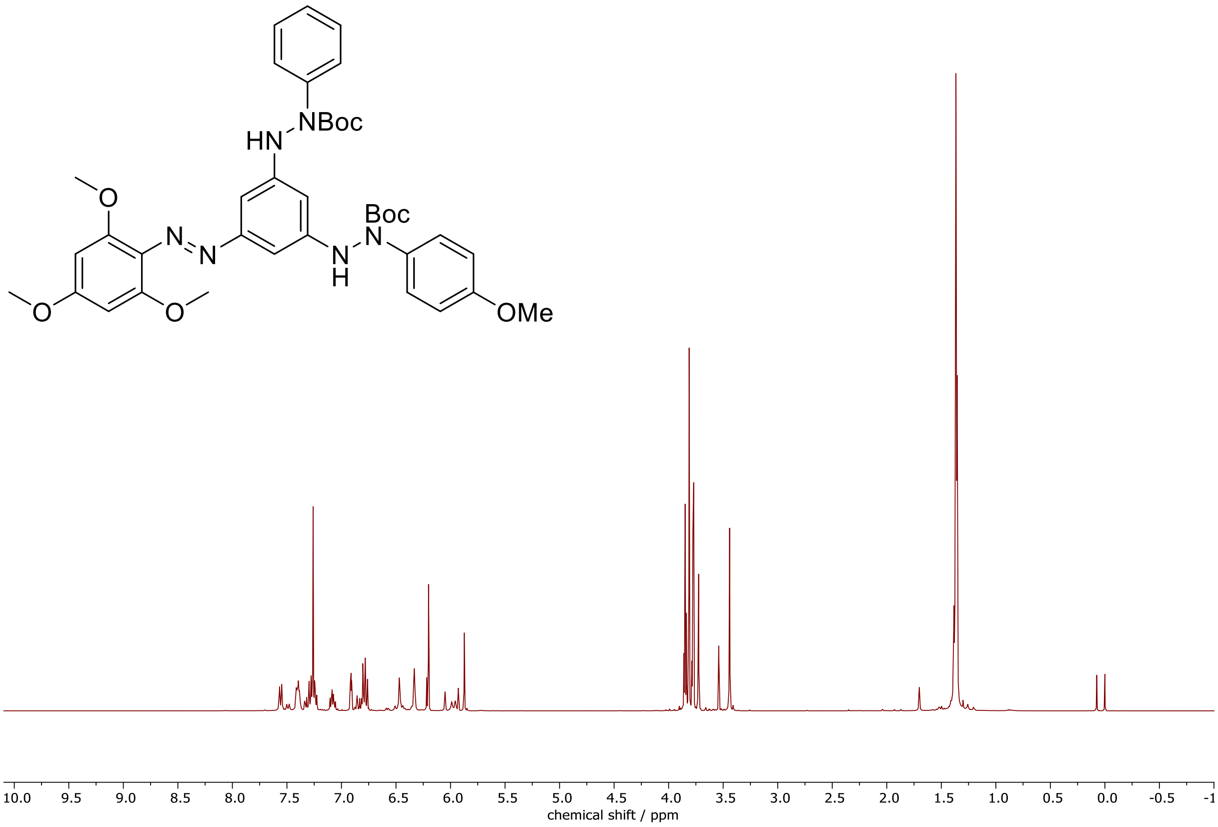

(MeO)3-Ph-CN-trisazobenzene 3a

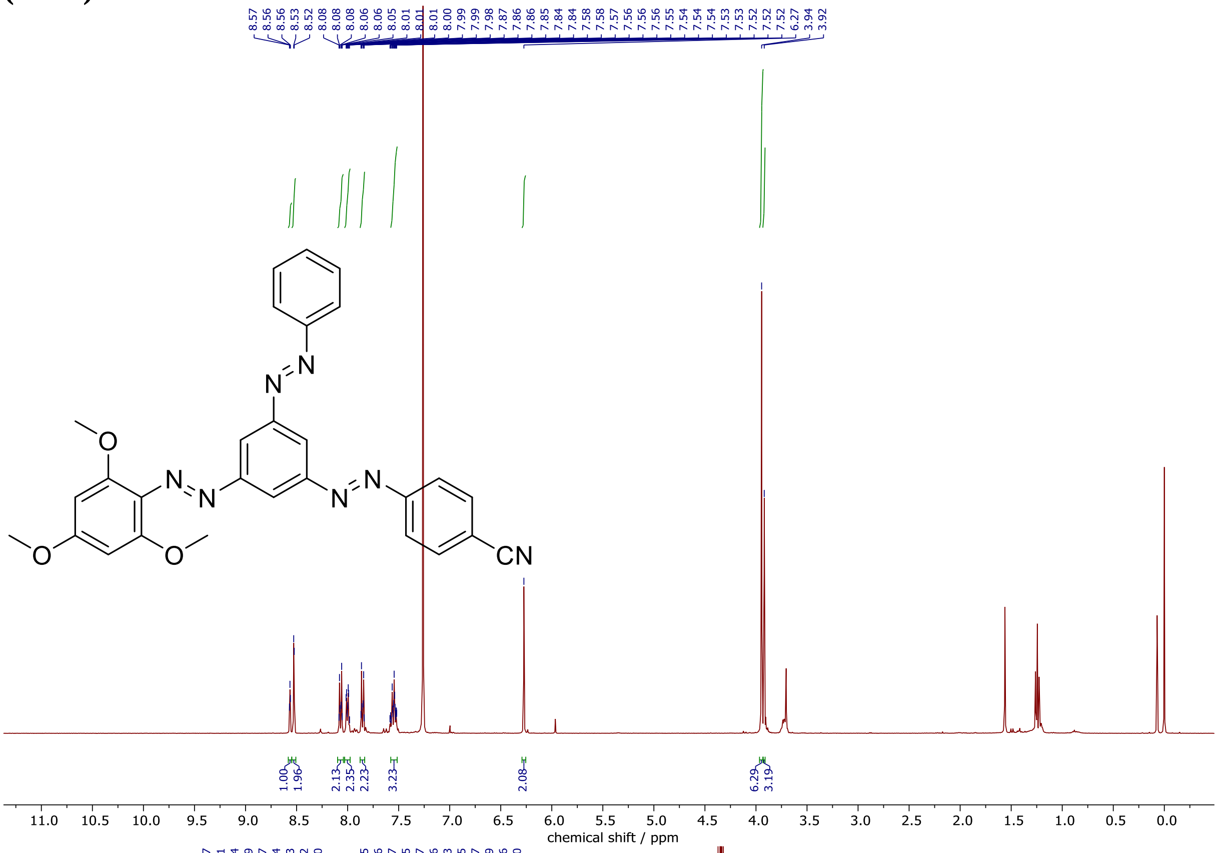

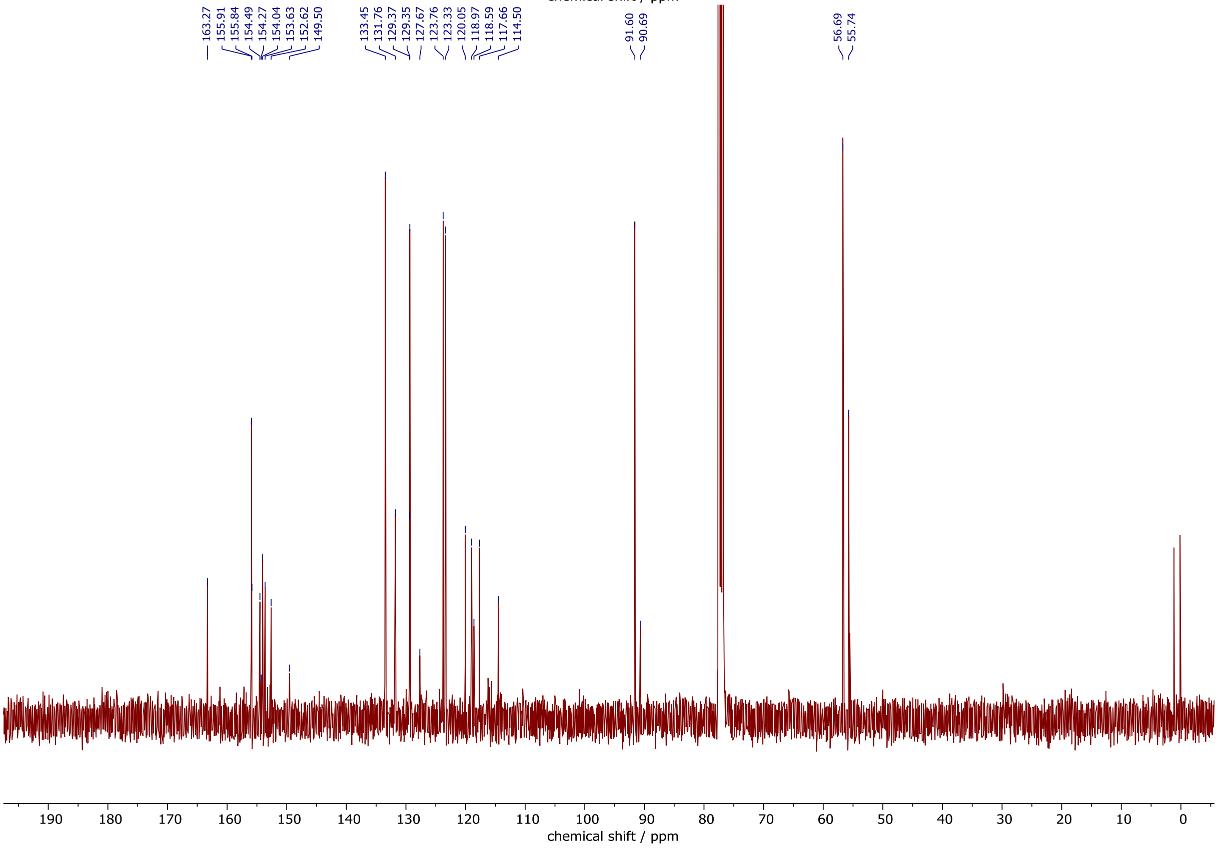

(MeO)$_3$-Ph-OMe-trisazobenzene 3b

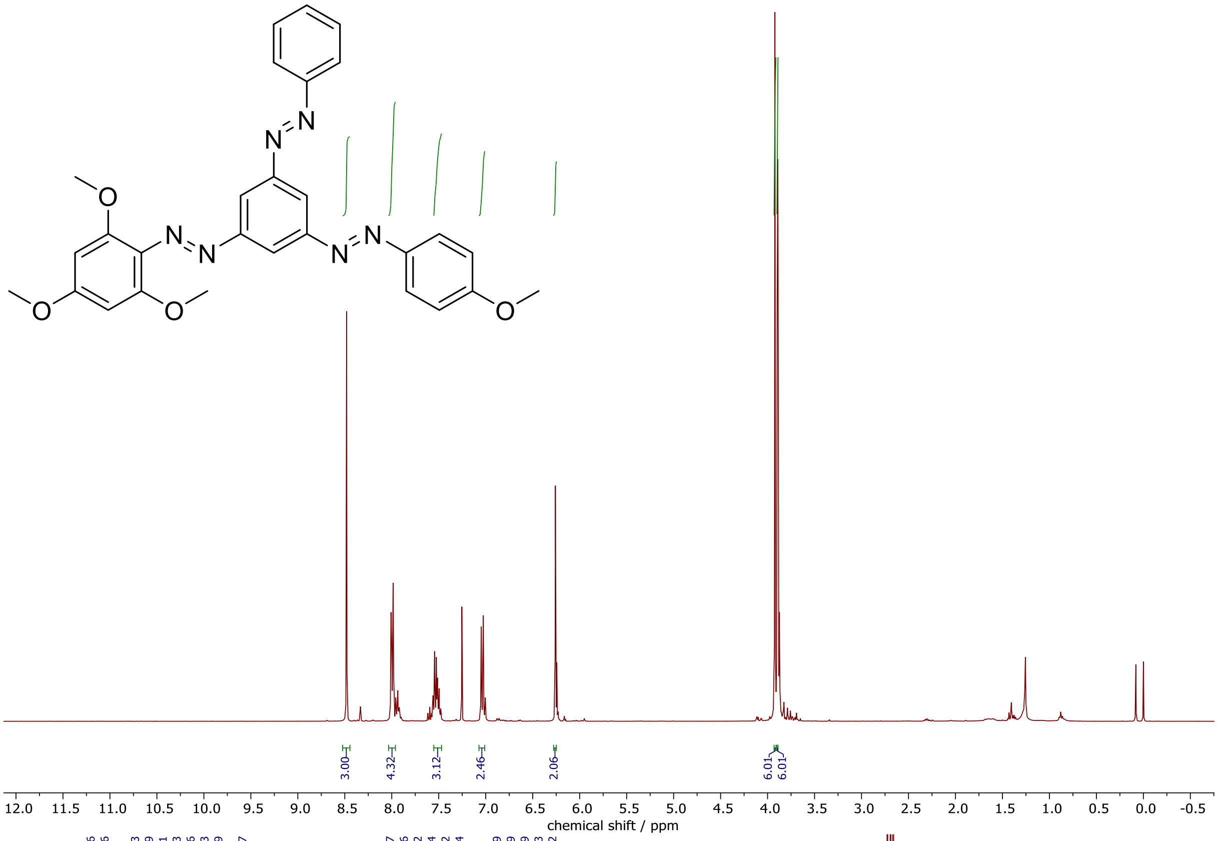

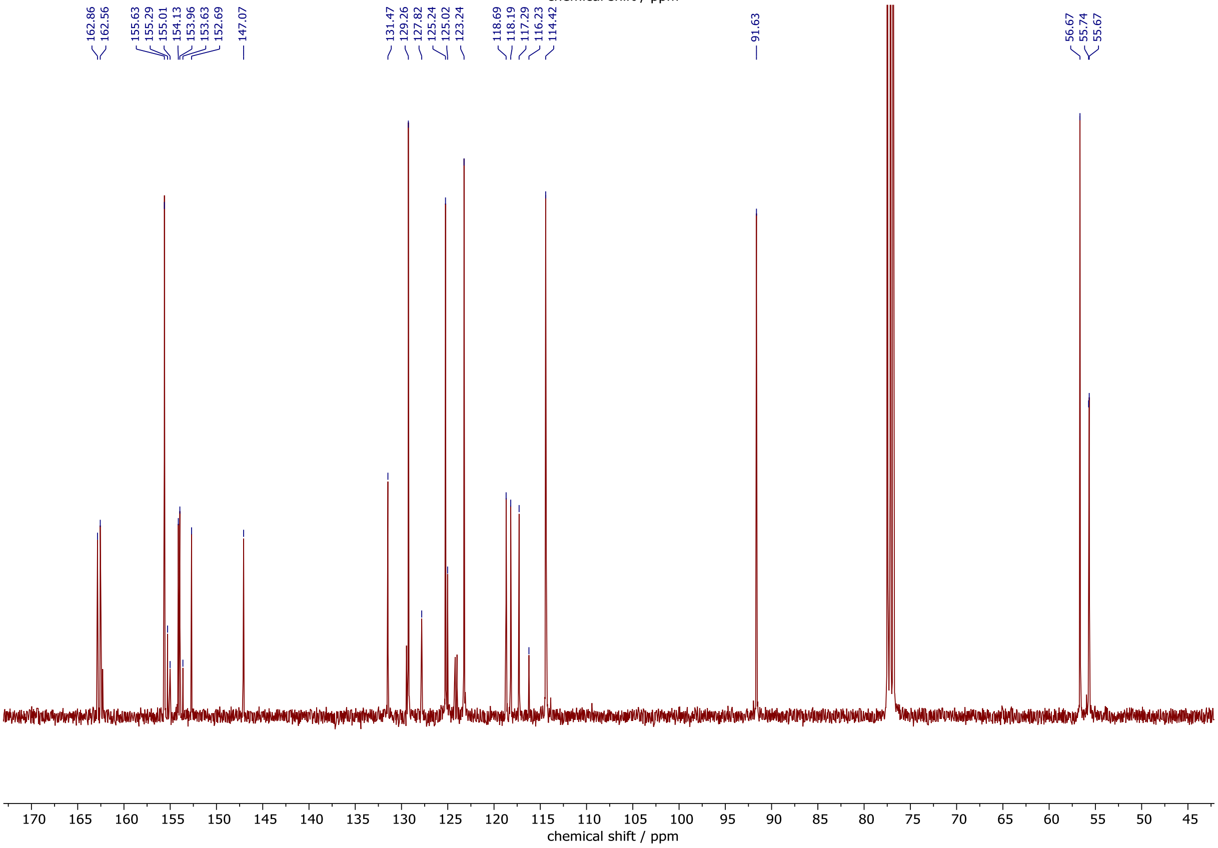

References

1. Jiang, L.; Lu, X.; Zhang, H.; Jiang, Y.; Ma, D. *J. Org. Chem.* **2009,** *74* (12), 4542–4546. doi:10.1021/jo9006738
2. Wang, Z.; Skerlj, R. T.; Bridger, G. J. *Tetrahedron Lett.* **1999,** *40* (18), 3543–3546. doi:10.1016/S0040-4039(99)00561-4
3. Wolter, M.; Klapars, A.; Buchwald, S. L. *Org. Lett.* **2001,** *3* (23), 3803–3805. doi:10.1021/ol0168216
4. Bílek, P.; Slouka, J. *J. Heterocycl. Chem.* **2002,** *39* (2), 357–361. doi:10.1002/jhet.5570390219
5. Priewisch, B.; Rück-Braun, K. *J. Org. Chem.* **2005,** *70* (6), 2350–2352. doi:10.1021/jo048544x
6. Anwar, N.; Willms, T.; Grimme, B.; Kuehne, A. J. C. *ACS Macro Lett.* **2013,** *2* (9), 766–769. doi:10.1021/mz400362g
